# siRNA-loaded folic acid-modified TPGS alleviate MASH *via* targeting ER stress sensor XBP1 and reprogramming macrophages

**DOI:** 10.7150/ijbs.96113

**Published:** 2024-07-08

**Authors:** Manman Zhu, Yong Cheng, Li Zuo, Bao Bin, Haiyuan Shen, Tao Meng, Zihao Wu, Peng Rao, Yue Tang, Shuojiao Li, Honghai Xu, Guoping Sun, Hua Wang, Guiyang Zhang, Jiatao Liu

**Affiliations:** 1 Department of Pharmacy, the First Affiliated Hospital of Anhui Medical University, Hefei 230022, Anhui Province, China.; 2 School of Pharmacy, Anhui Medical University, Hefei 230032, Anhui Province, China.; 3 Department of Pathology, Laboratory of Mucosal Barrier Pathobiology, Anhui Medical University, Hefei230032, Anhui, China.; 4 Boston Children's Hospital, Harvard Medical School, Boston, MA 02115, United States.; 5 Department of Oncology, the First Affiliated Hospital of Anhui Medical University, Hefei 230022, Anhui Province, China.; 6 Department of Pharmacology, School of Basic Medical Sciences, Anhui Medical University, Hefei 230032, Anhui Province, China.; 7 Department of Pathology, The First Affiliated Hospital of Anhui Medical University, Hefei 230022, Anhui Province, China.; 8 Department of Pharmacy, Anhui University of Chinese Medicine, Hefei 230012, Anhui Province, China.

**Keywords:** Metabolic dysfunction-associated steatohepatitis, Endoplasmic reticulum stress, Macrophages, X-box binding protein 1, D-α-Tocopheryl polyethylene glycol 1000 succinate

## Abstract

Macrophages show high plasticity and play a vital role in the progression of metabolic dysfunction-associated steatohepatitis (MASH). X-box binding protein 1 (XBP1), a key sensor of the unfolded protein response, can modulate macrophage-mediated pro-inflammatory responses in the pathogenesis of MASH. However, how XBP1 influences macrophage plasticity and promotes MASH progression remains unclear. Herein, we formulated an *Xbp1* siRNA delivery system based on folic acid modified D-α-tocopheryl polyethylene glycol 1000 succinate nanoparticles (FT@XBP1) to explore the precise role of macrophage-specific *Xbp1* deficiency in the progression of MASH. FT@XBP1 was specifically internalized into hepatic macrophages and subsequently inhibited the expression of spliced XBP1 both *in vitro* and *in vivo*. It promoted M1-phenotype macrophage repolarization to M2 macrophages, reduced the release of pro-inflammatory factors, and alleviated hepatic steatosis, liver injury, and fibrosis in mice with fat-, fructose- and cholesterol-rich diet-induced MASH. Mechanistically, FT@XBP1 promoted macrophage polarization toward the M2 phenotype and enhanced the release of exosomes that could inhibit the activation of hepatic stellate cells. A promising macrophage-targeted siRNA delivery system was revealed to pave a promising strategy in the treatment of MASH.

## 1. Introduction

The incidence of nonalcoholic fatty liver disease (NAFLD) is steadily increasing worldwide due to excessive nutritional intake and lack of exercise, which seriously affects the health of one-quarter of the world's population^1^. Approximately 25% of NAFLD patients with simple steatosis evolve into metabolic dysfunction-associated steatohepatitis (MASH; previously named nonalcoholic steatohepatitis, NASH) featured as liver fibrosis, inflammatory infiltration, and lobular lesions^2^, and more than 20% of patients suffer from a progression to liver fibrosis or even cirrhosis^3^. Scientists have made enormous efforts to develop effective interventions for NAFLD patients; however, no drugs have been approved for the treatment of MASH^4^.

A major pathological feature of MASH is the infiltration of pro-inflammatory cells^5^, such as macrophages that are essential for immune activation in the initiation and progression of MASH^6^. When there are environmental lipid signals, hepatic macrophages secrete pro-inflammatory factors and promote the development of steatohepatitis. Some researchers have also reported that the recruitment of macrophages and subsequent cytokine release initiate inflammatory cascades and activate hepatic stellate cells (HSCs), the most dominant hepatic fibrogenic cell population, which further promote MASH progression^7^, ^8^. Hepatic macrophages and HSCs are often observed near the injured area, which enables the exchange of cytokine signals and perpetuates inflammation and fibrosis^9^. Moreover, macrophage-derived transforming growth factor β-1^10^ or signaling molecules released by injured hepatocyte mitochondria^11^, ^12^ directly activate HSCs, upregulate alpha-smooth muscle actin (α-SMA), and aggravate extracellular matrix accumulation. Given the great plasticity of macrophages and their importance in tissue remodeling and immunity via interaction with HSCs, determining the different schema of macrophage-HSC interaction has received increasing attention from researchers and may be suitable targets for MASH treatment.

Excessive accumulation of fatty acids and disordered lipotoxic metabolites are key events in the initiation of NAFLD^13^. Studies have also shown that sustained exposure to lipotoxic fatty acids can cause hepatocyte injury by inducing endoplasmic reticulum (ER) stress and subsequently triggering the unfolded protein response (UPR) pathway^14^, ^15^. The pathophysiology of lipotoxicity-related ER stress is highly relevant to the ballooning degeneration of hepatocytes^16^ and aggravating inflammation and fibrosis in the liver of patients with MASH^17^, ^18^. Although the role of ER stress in promoting the transition from NAFLD to MASH has been demonstrated in multiple mouse models^19^, its origin and mechanism remain largely unclear. IRE1α-XBP1 is the most conservative pathway among the three UPR branches, and X-box binding protein 1 (XBP1) is a substrate of mRNA-splicing endonuclease IRE1α^20^. Recently, some studies demonstrated that XBP1s, a key transcription factor during ER stress, plays an irreplaceable role in regulating fibrogenesis by activating HSCs^21^. A further study indicated that the severity of liver pathology significantly correlated with the XBP1s levels in MASH patients. Moreover, hepatocyte- and hepatic macrophage-specific XBP1 knockout reduced hepatic inflammatory foci, downregulated fibrosis indices, and improved MASH, respectively^22^. These results revealed that XBP1-dependent ER stress plays an important role in MASH progression and therapeutic intervention to restore the ER balance, which paves a potential way to treat MASH.

Modulating ER stress in macrophages is a great challenge owing to the lack of a hepatic macrophage delivery system. The surface of activated macrophages overexpresses folate receptors compared with normal cells^23^, suggesting that nanocarriers with folic acid (FA) can be decorated to target and regulate these macrophages. D-α-Tocopheryl polyethylene glycol 1000 succinate (TPGS) is a water-soluble derivative of natural Vitamin E and formed by the esterification of Vitamin E succinate with polyethylene glycol (PEG) 1000, which is used for chemical coupling with polymers, and has been certified as a safe pharmaceutical adjuvant by the US Food and Drug Administration (FDA). TPGS have both hydrophilic and hydrophobic groups and significantly improve the stability and permeability of drugs, e.g., decorative a small interfering RNA (siRNA)^24^. Unmodified siRNAs are a class of polyanionic hydrophilic molecules with low membrane permeability that are not easily taken up by cells. In addition, siRNAs can be hydrolyzed by nucleases *in vivo*, having a short half-life. Polymeric carriers are delivery systems that can overcome these two obstacles^25^. These suggest that folic acid-modified TPGS (FA-TPGS) can be used to encapsulate siXBP1s to target hepatic pro-inflammatory macrophages.

Herein, we prepared hybrid FA-TPGS by a simple cross-linking action for stable and effective delivery of siRNA to specifically knockdown XBP1 (named FT@XBP1) in hepatic macrophages. Subsequently, the safety and therapeutic potential of FT@XBP1 were studied in advanced MASH. Our results showed that polymer-siRNA hybrid nanocarriers were capable of alleviating dietary-induced steatohepatitis by safely and effectively disturbing the ER stress sensor XBP1s and reprogramming macrophages toward the M2 phenotype. This work paves a novel way for reversing MASH progression.

## 2. Materials & methods

### 2.1 Fabrication of FT@XBP1

Rhodamine B (RhB) isothiocyanate DMSO solution (0.266 mg/mL, 500 uL) was added into the si*Xbp1* enzyme-free aqueous solutions (1.65 mg/mL,1 mL, n(RhB): n(si*Xbp1*) = 2: 1) and shaken well. NaHCO_3_ solution (0.1 mL, 1 M) was added to adjust pH to 8.0 and then stirred overnight. The mixture was dialyzed using deionized H₂O (9 L, 3 times) and lyophilized to obtain rhodamine B modified si*Xbp1* (RhB-si*Xbp1*), and then stored at -20 °C. Folic acid modified TPGS delivery system was prepared as previously reported^26^. Briefly, the functional FA-TPGS@RhB-si*Xbp1* (herein called FT@XBP1) was synthesized by the cross-linking effect (n(FA-TPGS): n(si*Xbp1*) = 5: 1). Firstly, FA-TPGS (2.24 mg), RhB-si*Xbp1* and 1,4 dioxane (2 mL) were added into a penicillin bottle. The samples were sonicated for 20 min and then assembled using a micro flow meter (7 mL ultrapure water, 2 h with rapid stirring). And the nanocarriers were dialyzed after assembly. Then products were lyophilized for 24 h and stored at -20 °C for further characterizations.

### 2.2 Characterization of FT@XBP1

Details of cell culture are presented in the supplementary materials. Firstly, the morphologies of TPGS and FT@XBP1 incubated with / without RAW 264.7 cells were visualized via Talos L120C G2 transmission electron microscopy (TEM, Thermo, USA) with an accelerating voltage of 300 kV. The particle size was determined using a Zeta sizer Nano-ZS (Malvern ZS90, UK) . The UV-Vis absorption measurement was identified by UV spectra (Shimadzu, UV-1900, Japan). The FT-IR characterization absorption was recorded on Fourier transform infrared spectrum (FTIR, PerkinElmer Spectrum Frontier, USA) and characteristic fluorescence absorption was imaged by RF5301PC fluorescence spectra (Shimadzu, Japan).

### 2.3 Serum stability and relative release

For detecting the stability of FT@XBP1 in serum, FT@XBP1 and unmodified si*Xbp1* were incubated with serum for 0, 2, 4, 6, 12 and 24 then nucleic acid gel electrophoresis was used to detect the serum stability of FT@XBP1 and si*Xbp1*. For detecting the releasing of RhB-si*Xbp1*, decorative FT@XBP1 nano-carriers were dispersed in 1mL PBS (pH 5.0 or pH 7.4) and placed in a dialysis bag with an MWCO15000 (9 L) at room temperatures. The concentration of RhB-si*Xbp1* was measured by Infinite M1000 PRO full-wave length enzyme labeler (Tecan, Switzerland) at a series of times (0, 10, 20, 30 and 40 h).

### 2.4 Macrophages uptake FT@XBP1

FT@XBP1 were cocultured with RAW 264.7 or mTHP-1 cells stimulated with lipopolysaccharide (LPS) or IL-4 + IL-13 for 24 h, respectively. Then cells were washed and sequentially added 4% paraformaldehyde for 5-10 min, and co-incubated with 5% BSA at room temperature for 1 h. Whereafter, RAW 264.7 and mTHP-1 cells were co-incubated with F4/80 and CD68, respectively. Nuclei was stained with DAPI for 10 min. The images were captured by confocal laser scanning microscope (Zeiss, LSM800+ airyscan, Germany).

### 2.5 The impacts of FT@XBP1 on cell viability, apoptosis and the expression of XBP1s

For measuring cell viability, RAW 264.7 cells were seeded in 96-well plates (5 × 10^3^ cells/well), and incubated with a range of concentrations of FT@XBP1 nanocarriers (*c*(FA-TPGS): 0, 0.15, 0.3, 0.6, 1.2, 2.4, 4.8 μg/mL) for 48 h. Cell counting kits 8 reagents (Target Mol, Boston, USA) were added and co-incubated for 2-4 h, and the absorbance was measured at 450 nm using enzymatic labels (BioTeck, USA). To investigate apoptosis and the expression of XBP1s, cells were treated with FT@XBP1 nanocarriers and relative controls for 48 h. Then cells were collected, for measuring apoptosis, cells were stained with Annexin V and PI solution according to the manufacturer's instructions (Bestbio, Nanjing, China), and data was obtained by CytoFLEX (Beckman, USA), while cells were collected and analyzed by Western blot assay for detecting the expression of XBP1s.

### 2.6 Patients and animal models

Normal (N = 22) or steatosis (N = 27) liver tissues were obtained during liver transplantation procedures at the First Affiliated Hospital of Anhui Medical University between 2018 and 2021. Male C57BL/6J mice at the age of 5-7 weeks (18-20 g) were purchased from Gempharmatech corporation (Jiangsu, China). Mice were housed under specific pathogen free (SPF) and fed with either a chow diet (CD) or the fat-, fructose- and cholesterol-rich (FFC) diet for up to 24 weeks^18^. Mice in FFC group were randomly divided into three subgroups (FFC, FFC + FT@NC, FFC + FT@XBP1) at the beginning of 20 weeks. FT@XBP1 and FT@NC were injected intravenously (2 OD, 0.05 nM, 100 μL) every three days for 9 times through the tail vein. Food intake and body weight were recorded once a week. The blood samples and tissues were harvested from mice, and the livers and epididymal fat were rapidly excised and weighed, then the livers were snap frozen and stored at -80°C for subsequent analysis. For determining the specificity of adeno associated virus (AAV)-8 on hepatic macrophages, C57BL/6J mice were transduced intravenously with 100 μL (1 × 10^12^ copies / mL) of AAV-8 (Hanbio, Shanghai, China) as the manufacturer's Instructions.

### 2.7 Small animal imaging

Kunming mice (18-20 g) were purchased from Anhui Provincial Laboratory Animal Center and injected intravenously with FT@NC and FT@XBP1 through tail vein. The small animal imaging system (Spectral Instruments Imaging, Mix, USA) was used to measure NIR distributions in mice after injection for 2, 6, 12, 24, 48 and 72 h (excitation, 500 nm; emission, 630 nm). Tissues such as heart, liver, spleen, lungs and kidneys were collected for further analysis 72 h after the last injection.

### 2.8 Biochemical assays

Blood samples were taken and serum was collected (3000 rpm, 5 min) at room temperature for determination of alanine aminotransferase (ALT), aspartate transaminase (AST), total cholesterol (TC) levels. The level of AST and ALT of serum were detected by Mindray BS-430 automatic biochemistry analyzer. Liver tissue was collected snap frozen and stored at -80 °C for hepatic total triglycerides (TG) levels. The quantities of hepatic total triglycerides (TG) in the livers and the level of serum TC were assayed according to the manufacturers' protocols (ADS Bio, Jiangsu, China).

### 2.9 Histological analysis

Steatosis was evaluated by H&E and Oil Red O stained liver sections according to the manufacturer's instructions. Briefly, liver tissues were fixed with 4% paraformaldehyde and dehydrated gradually with ethanol (70-100%), and then put on paraffin. The tissues were incised at 4 μm thick by a rotary microtome (Thermo, USA) for hematoxylin and eosin (H&E), masson and sirius red staining. For analysis hepatic steatosis, 5-10 μm thick frozen liver sections were embedded with OCT and employed for Oil red O staining.

Hepatic steatosis and hepatocellular ballooning lesions were analyzed according to NAFLD Activity Score (NAS). Grading systems (G0-G4) have been developed to characterize the histological inflammation changes in NAFLD. The degree of liver fibrosis was determined through the Scheuer scoring system, and the description from S0 to S4 represents the aggravation of liver fibrosis^27^. Mice were grouped into significant liver fibrosis when the stage was greater than or equal to S2, while greater than or equal to S3 was classified as progressive fibrosis.

### 2.10 Immunohistochemistry (IHC) and immunofluorescence (IF)

Sections of 4 μm paraffin-embedded liver tissues were prepared for IHC tests, while frozen sections of liver tissues were used for IF analysis. For IHC tests, the slides were dewaxed, hydrated, permeated, and immersed in 5% BSA solution, and incubated with antibodies against F4/80 and α-SMA overnight. Then the sections were washed and co-incubated with a biotinylated secondary antibody and a peroxidase-conjugated streptavidin. Finally, the sections were visualized using diaminobenzidine and counterstained using Digital Slide Scanner (Pannoramic MIDI, Dalian, China).

For immunofluorescence experiments, various primary antibodies (dilution 1: 200) against XBP1s, F4/80, CD86, CD163, CD11b and iNOS were co-incubated with frozen sections of liver tissues overnight, followed by incubating with a corresponding secondary antibody. Subsequently, the cells were reacted with DAPI and imaged by Digital Slide Scanner viewed at × 10 and/or × 40 magnification.

For fluorescence analysis the impact of FT@XBP1 on HSCs activation, JS-1 cells were treated with FT@XBP1 and sequentially fixed with 4% paraformaldehyde and permeabilized with 0.5% Triton X-100. After immersed in 5% BSA for 1 h, the coverslips were incubated with antibodies against α-SMA and Col1αI (dilution 1: 200) and relative secondary antibody (dilution 1: 50), respectively. Finally, DAPI was used for staining nuclear, and the sections were viewed by fluorescence (Leica, German) or confocal microscopy.

### 2.11 Isolation of primary liver macrophages

The mice were anesthetized with 1% isopentobarbital sodium, and the abdominal cavity was opened to separate the inferior vena cava and hepatic portal vein. The hepatic portal vein was cut immediately following intubation of the inferior vena cava, then 1 × Hanks solution was poured over the liver for 5 min to remove residual blood and reduce the concentration of calcium ions between the hepatocytes, followed by continuous perfusion of 50 mL collagenase I solution (Sigma) preheated to 37 °C. The liver digests were filtered through a cellular filter and washed with Gey balanced salt solution (Sigma, G9779) containing DNase I (2 mg/mL, Roche Diagnostics). The liver macrophages were isolated by discontinuous density gradient precipitation in percoll at 25% and 50% concentrations. The cells were further analyzed by flow cytometry.

### 2.12 Flow cytometry and enzyme-linked immunoassay (ELISA)

Primary hepatic macrophages were labeled with FITC-F4/80, APC-CD86, PE-CD163 antibodies (Biolegend, USA). While the phenotypes transformation of RAW 264.7 cells were measured by co-incubating with primary antibodies FITC-F4/80, APC-CD86 and PE-CD163 after treating with PA and/ or FT@XBP1 for 48 h. And the data were obtained by flow cytometer (Beckman, USA).

The expression level of IL-10, IL-6, IL-1β and TNF-α in the supernatants of PA and/or FT@XBP1 treated RAW 264.7 cells and the serum of mice were analyzed by ELISA kits (Mmbio, Jiangsu, China).

### 2.13 Exosome isolation, identification and incorporation

RAW 264.7 cells were cultured in DMEM medium containing 10% FBS. When the confluence was about 60%, the original medium was discarded and replaced with medium containing 10% exosome-free FBS, and treated with PA and/or FT@XBP1. Then the supernatants were collected, and exosomes were extracted using ExoQuick-TC precipitation kits (System Bioscience, CA) according to manufacturer's instructions. The purified exosomes were resuspended in PBS according to the concentration of exosomal-proteins using a BCA protein quantitation kit (Thermo Fisher, America), and stored at -80 °C.

TEM analysis was carried out to check whether the isolated vesicles have a typical morphology of exosome. Briefly, 10 μL of purified exosomes were added to the copper mesh, and then stained with 2% tungsten phosphate for 3 min. Finally, the grids were washed with PBS and air-dried at room temperature. Representative images were taken at 80 kV using a transmission electron microscope (Talos L120C G2, Thermo, USA). For detecting exosomal-proteins, Western blot analysis was employed as previously reported^28^.

JS-1 cells were co-incubated with PBS or PKH67 (Sigma-Aldrich, USA) -labeled exosomes for 12 h, then cells were washed and fixed with 4% paraformaldehyde (Solarbio, China) and penetrated with 0.1% Triton X-100 (Solarbio, China). Finally, cells were stained with DAPI, and the sections were observed under a laser scanning confocal microscope (Leica, German).

### 2.14 The impact of macrophage-derived exosomes on JS-1 cells

The exosomes derived from RAW 264.7 cells pretreated with/ without PA (Exo-con, Exo-PA, Exo-FA-TPGS, Exo-FT@NC and Exo-FT@XBP1) (10 μg/mL) were incubated with JS-1 cells and the indicators of HSCs activation were monitored by WB, qRT-PCR and immunofluorescence.

### 2.15 Western blot analysis

Macrophages (RAW 264.7 or mTHP-1 cells) or murine hepatic stellate cell lines JS-1 were treated with agents, such as palmitic acid (PA), tunicamycin (TM) or FT@XBP1 and relative control according to the experiment designs. Then, the exosomes or cells were collected and lysed with protein lysate (RIPA: PMSF = 100: 1), and the total proteins were quantified by a BCA protein quantitation kit (Thermo Fisher, America). Proteins were separated by an SDS-PAGE gel and then transferred to the PVDF membrane (Millipore, Bedford, MA, USA). Then the membrane were incubated with various primary antibodies overnight, the details of antibodies were listed in **Supplementary Table S1**. The corresponding secondary antibodies (1: 10000 dilution) were incubated sequentially at 37 °C for 1 h. Finally, the bands were photographed using an Image Quant™ LAS-4000 Mini Developer (Fuji, Japan), and the results were semi-quantitatively analyzed using Scion Image (version 4.0.3.2).

### 2.16 Real-time quantitative polymerase chain reaction (qRT-PCR) assays

Total RNA was extracted from cells and liver tissues using TRIzol according to the manufacturer's instructions. The reverse transcription procedure referred to Evo M-MLV RT kit instructions and the qRT-PCR experimental details as the guideline of SYBR®Green Premix Pro Taq HS qPCR Kit II (Accurate Biotechnology (Human) Co., Ltd., Hunan, China). The rest of the details are presented in the supplementary materials. The primer sequences used in the current work were shown in **Supplementary Table S2**. The expression levels of target gene were normalized using β-Actin.

### 2.17 Statistical analysis

Data are expressed as the means ± SD and were analyzed using SPSS 17.0 software (SPSS Inc., Chicago, Illinois, USA), each experiment was carried out in triplicate independently. Two-tailed t test was used to measure the statistical difference between the two groups, and one-way ANOVA were used for three or more groups and followed by Tukey's post hoc test. *P* < 0.05 were considered significant.

## 3. Results

### 3.1 XBP1s is upregulated in macrophages in high-fat-induced ER stress models

**Figure 1:**
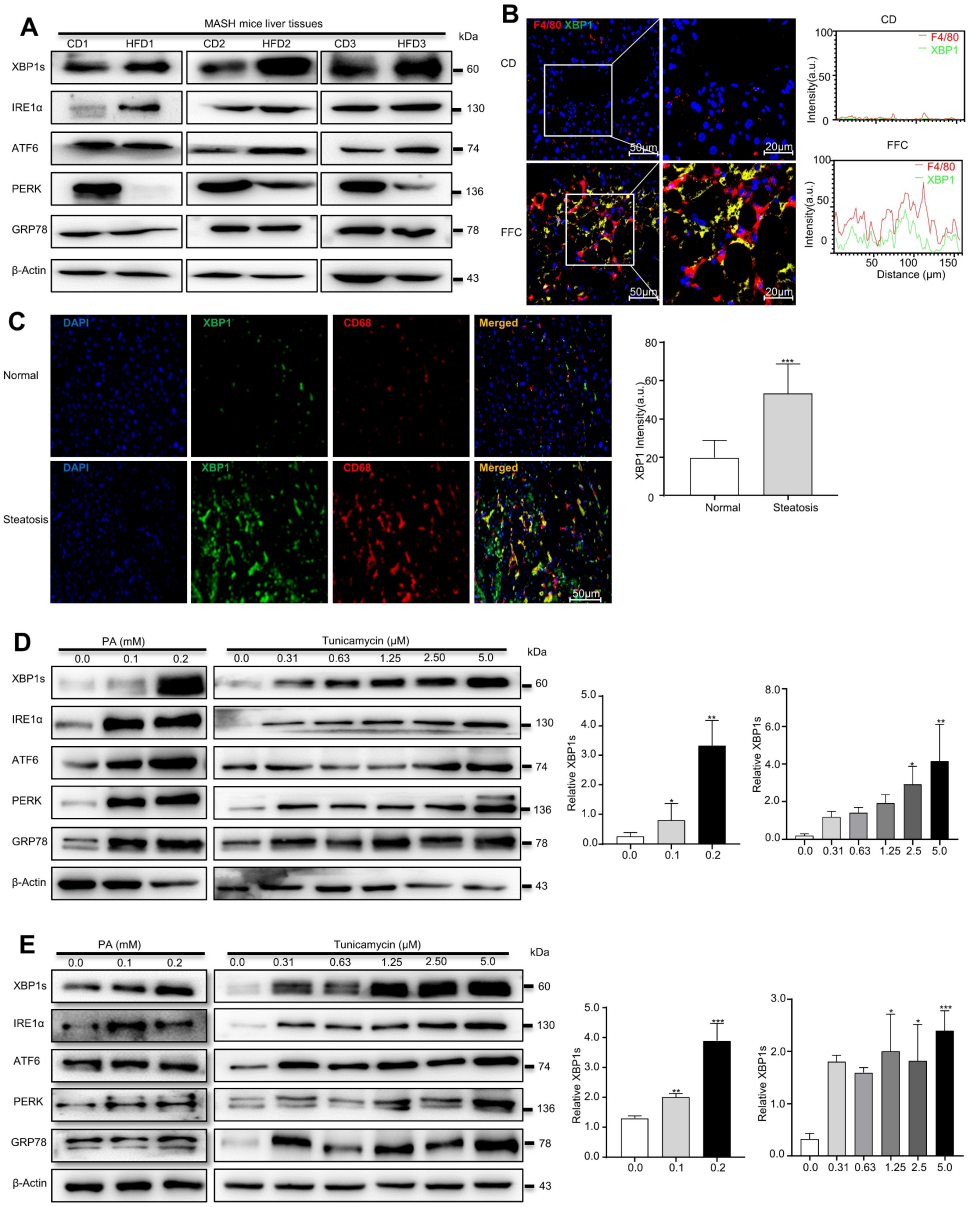
** XBP1s is increased in macrophages in high-fat induced ER stress model.** (A) The expression of XBP1s, ATF6, IRE1α,GRP78 and PERK in HFD diet induced MASH mice was determined by Western blot analysis(n = 3). Immunofluorescence images of liver tissues of FFC diet-fed mice (B) and NAFLD patients (C) dual stained with XBP1 (green channel) and F4/80 or CD68 (red channel), and semi-quantitative analyzed (scale bar = 50/20 μm). (D-E) The expression of XBP1s, IRE1α, ATF6, GRP78 and PERK in palmitic acid- and TM-treated RAW 264.7 (D) and mTHP-1 cells (E), and semi-quantitatively analyzed the band intensity of XBP1s. Data are presented as the means ± SD (error bar) of at least three independent experiments. *P < 0.05, **P < 0.01, ***P < 0.001 compared with relative controls.

A recent study demonstrated that XBP1 was upregulated in both liver cells and macrophages in the liver tissues of MASH patients^22^. To explore the precise role of XBP1 in MASH progression, the ER stress sensor levels were determined using Western blot analysis, and the expressions of GRP78, ATF6, IRE1α, and XBP1s were significantly overexpressed in the livers of high-fat diet (HFD)-fed mice (18 months) compared with chow diet (CD)-fed mice, whereas protein kinase R-like ER kinase (PERK) was downregulated in HFD-fed mice (Fig. 1A and Fig. S1A). XBP1s, a key ER stress-related transcription factor, was most significantly elevated compared to other ER stress markers (Fig. S1A). Moreover, immunofluorescence (IF) assays confirmed that the expression of XBP1 in the liver tissues of fat-, fructose- and cholesterol-rich (FFC) diet-fed 24-week-MASH mice was higher than in CD-fed mice (Fig. S1B), suggesting that XBP1s may play an essential role in the process of MASH progression. Furthermore, immunofluorescence showed that the expression of XBP1 was significantly increased in F4/80 positive cells in the liver tissue of FFC diet-fed mice compared with the CD group (Fig. 1B). In addition, we found that XBP1 was highly expressed in CD68-positive macrophages in liver of MASH patients (Fig. 1C). These results suggest a relationship between the macrophages and XBP1-dependent ER stress in the pathology of MASH.

Palmitic acid (PA) were used to treat macrophages *in vitro* and establish high-fat models. In agreement with the *in vivo* results, PA (0.1 mM, 0.2 mM) treatment increased the expression levels of UPR sensors (ATF6, IRE1α, and PERK and XBP1s) in RAW 264.7 cells (left panel in Fig. 1D and Fig. S1C), and XBP1s increased most significantly. Subsequently, the *in vitro* cell culture system was optimized to mimic the ER stress conditions by coculturing RAW 264.7 cells with a series of TM solutions (0.31-5.0 μM). TM dose-dependently upregulated ER stress-related proteins, and the treatment with 2.5 μmol/L of TM for 24 h was considered the optimal scheme (right panel in Fig. 1D and Fig. S1D). We also treated human acute monocytic leukemia cell THP-1 derived macrophages (mTHP-1) with PA or TM, and we found that both PA (left panel in Fig. 1E and Fig. S1E) and TM (right panel in Fig. 1E and Fig. S1F) dose-dependently activated ER stress, and XBP1s increased the most significantly. Taken together, our results confirmed that XBP1s might be a key transcriptional master regulator of ER homeostasis in hepatic macrophages; therefore, modulating XBP1s in hepatic macrophages may be a potential strategy to prevent MASH progression.

### 3.2 Preparation and characterization of FT@XBP1

First, we transiently transfected RAW 264.7 cells with small interference RNA (siRNA) of specific *Xbp1* and screened the optimal siRNA sequence (Fig. S2A) to clarify whether ER-stressed-hepatic macrophages promoted MASH progression in an XBP1s-dependent manner. Subsequently, we constructed an adeno-associated virus-8 (AAV-8) vector and administered through the tail vein of C57BL/6J mice, and found that AAV-8 (green) was mainly delivered to hepatocytes instead of F4/80+ hepatic macrophages (Fig. S2B). Furthermore, IRE1α inhibitor 4μ8C was not specific for XBP1, it inhibited XBP1 accompanying the low expression of IRE1α simultaneously (Fig. S2C). These results suggested that we should adopt other methods to accurately target hepatic macrophages.

**Figure 2:**
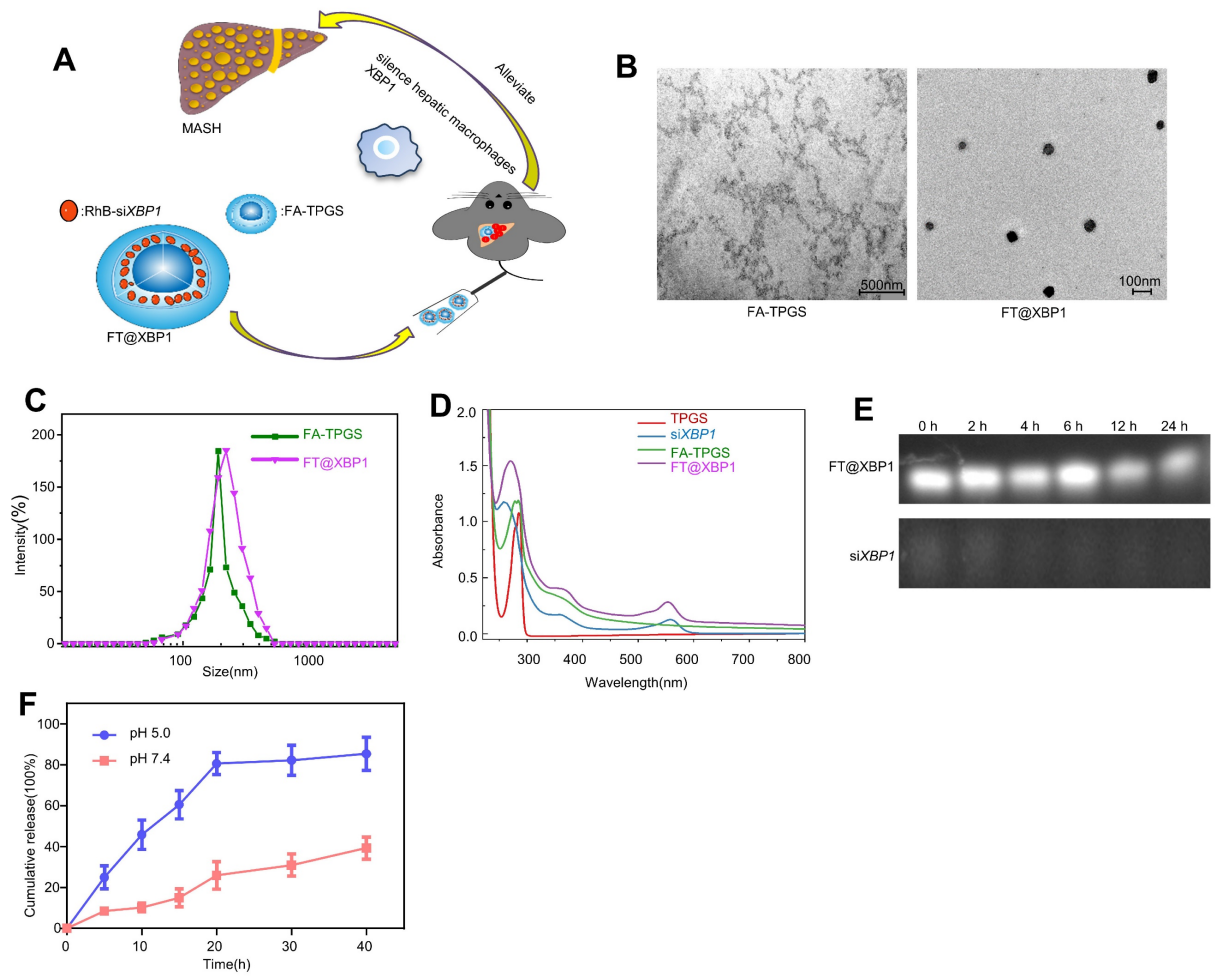
** Preparation and characterization of FT@XBP1.** (A)Scheme of the formation of folate-modified TPGS encapsulated with* Xbp1* siRNA (FT@XBP1) and its mechanism of action in hepatic macrophages. (B) Typical TEM observation of FA-TPGS and FT@XBP1 (scale bar = 500/100 nm). (C) The results of dynamic light scattering of FA-TPGS and FT@XBP1. (D) The UV spectra of TPGS, si*Xbp1*, FA-TPGS and FT@XBP1. (E) Serum stability of FT@XBP1 and si*Xbp1* at different time points (0, 2, 4, 12 and 24 h). (F) The cumulative drug release profile of FT@XBP1 under the conditions of pHs 7.4 and 5.0. Data are presented as the means ± SD (error bar) of at least three independent experiments.

TPGS nanocarriers in drug delivery systems have received widespread attention from researchers because they can enhance water-soluble drugs (e.g., siRNA) encapsulation, cellular uptake, and therapeutic efficacy^29^. Therefore, FA was chosen to modify TPGS nanomaterials (FA-TPGS) so that the encapsulated *Xbp1* siRNA can be more effectively delivered to hepatic macrophages in MASH (Fig. S3 and Fig. 2A). TEM and DLS indicated (Fig. 2B and Fig. 2C) that the particle size of si*Xbp1* encapsulated FA-TPGS (FT@XBP1) particle was 200 ± 15 nm with uniform morphology, similar to FT@XBP1 carriers. The characteristic peaks of FA-TPGS, TPGS and FT@XBP1 are at 220-300 nm. Both si*Xbp1* and FT@XBP1 showed small bulging ultraviolet absorption peaks at approximately 280 and 565 nm, respectively, indicating the successful binding of FA-TPGS carriers and RhB-si*Xbp1* (Fig. 2D). The infrared absorption peaks at 1,028, 1,590, and 3,300 cm^-1^ provide evidence for amide bond formation and demonstrate that FA and TPGS are combined. Furthermore, the typical -NH_2_ characteristic peak at 1,590 cm^-1^ and a clear splitting of the -COOH peak at 2,800 cm^-1^ appear after FA-TPGS nanomaterials encapsulated siRNA (FT@XBP1) or relative control (FT@NC) compared with the pure FA-TPGS (Fig. S4A). Fluorescence spectroscopy demonstrated that FT@NC and FT@XBP1 showed characteristic absorption peaks for rhodamine B at 550 nm (Fig. S4B). Ultraviolet, Fourier-transform infrared, and fluorescence spectroscopy characterization confirmed that FA-TPGS and RhB-siRNA have good interactions. FT@XBP1 nanomaterials degraded when cocultured with mouse serum for 24 h, while unwrapped si*Xbp1* degraded quickly (Fig. 2E). Fig. 2F shows that si*Xbp1* drugs were released at different pH levels, with a gradually stable release rate after 20 h. Furthermore, si*Xbp1* was released more rapidly at pH 5.0 than at pH 7.4, indicating that the nano-system continuously and quickly released the loaded drugs in the acidic microenvironment. The accumulative release of RhB-si*Xbp1* was up to 80% when FT@XBP1 nanomaterials were incubated in a phosphate-buffered saline solution (pH 5.0) for 20 h and remained unchanged at 40 h (Fig. 2F), suggesting that liver inflammatory microenvironment stimuli in MASH promote si*Xbp1* release from FT@XBP1 complexes. Collectively, these results demonstrated that FA-TPGS nanocarriers effectively encapsulated and released RhB-modified *siXbp1*.

### 3.3 FT@XBP1 was effectively incorporated into hepatic macrophages and inhibited the expression of XBP1s

**Figure 3:**
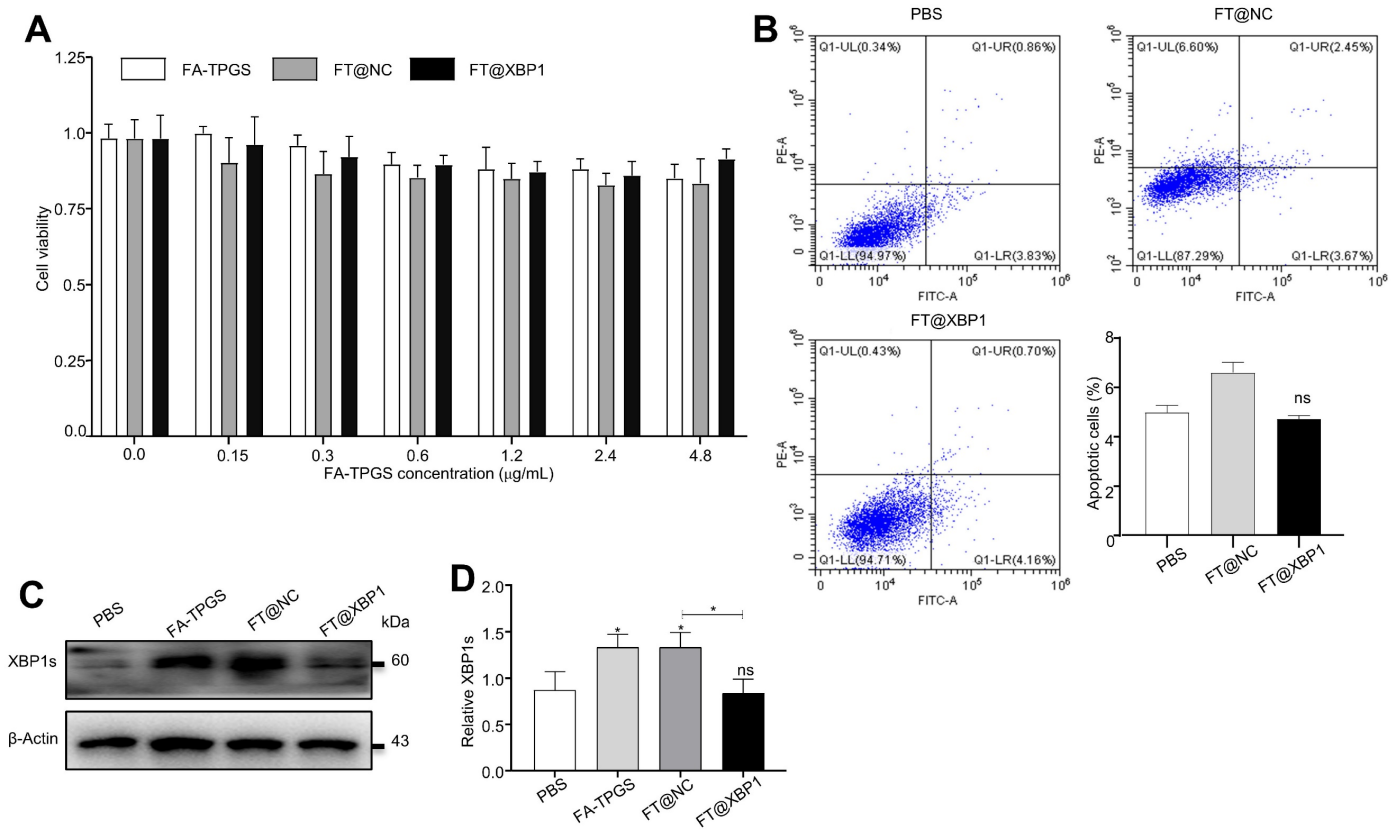
** Cytotoxic effects of FT@XBP1 on macrophages.** (A) Cell viability of FA-TPGS, FT@NC and FT@XBP1 on RAW 264.7 cells in a range of concentrations (0, 0.15, 0.3, 0.6, 1.2, 2.4 and 4.8 μg/mL) for 48 h. (B) Flow cytometry analysis determinate apoptotic and necrotic cells in RAW 264.7 cells after treated with FT@XBP1 at 2.4 μg/mL for 48 h, and quantitatively analyzed. The levels of XBP1s protein (C) in RAW 264.7 cells after co-incubating with FA-TPGS based nano-carriers for 48 h and (D) quantitatively analyzed. Data are presented as the means ± SD (error bar) of at least three independent experiments. *P < 0.05 compared with indicated groups.

Successful assembly of FA-TPGS nanocarriers-si*Xbp1* complexes urged us to test its safety *in vivo* and* in vitro*. First, FT@XBP1 nanocarriers were cocultured with macrophages *in vitro* to measure their effects on RAW 264.7 cells. We further explored the impact of FT@XBP1 on the cell viability and apoptosis and found that these FA-TPGS-based nanocarriers did not significantly reduce the cell viability (Fig. 3A) in RAW 264.7 cells at FA-TPGS concentrations of 0.15-4.8 μg/mL, while the concentration of 2.4 μg/mL had the best effect on XBP1 knockdown in RAW 264.7 cells (Fig. S4C and Fig. S4D). Subsequently, we stimulated RAW 264.7 cells with 2.4 μg/mL of FT@NC and FT@XBP1 for 48 h. Flow cytometric analysis showed that FT@XBP1 did not increase the percentage of apoptotic cells (Fig. 3B), but remarkably reduced the expression of XBP1s compared with the FT@NC group (Fig. 3D).

**Figure 4:**
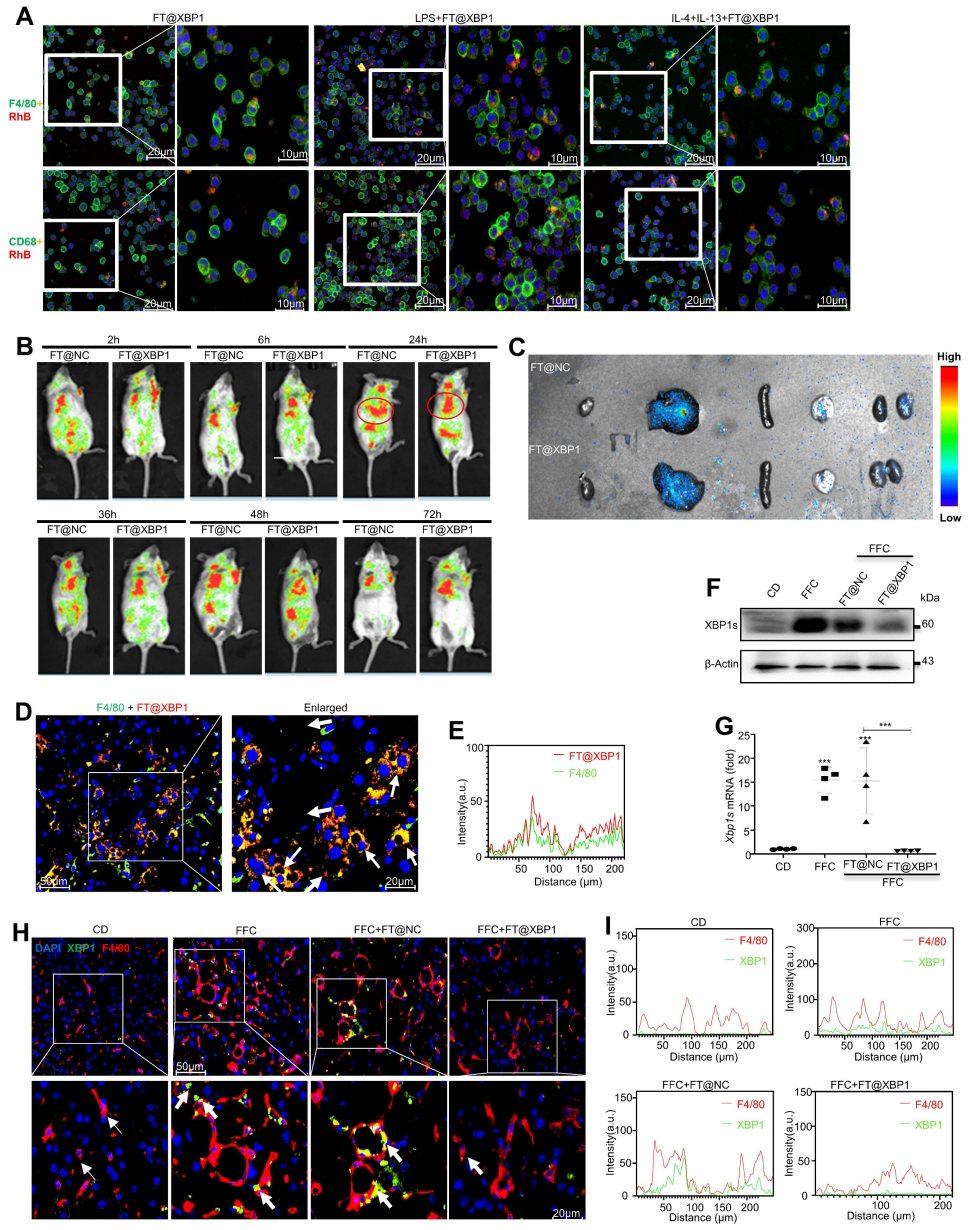
** FT@XBP1 specific targeted hepatic macrophage *in vitro* and *in vivo*.** (A) Representative images of internalization and accumulation of rhodamine B-labeled FT@XBP1 in RAW 264.7 (F4/80) and mTHP-1 (CD68) cells (scale bar = 20/10 μm). (B) *In vivo* NIR imaging of mice intravenously injected of FT@XBP1 and controls for 72 h. (C) Mean fluorescence intensities of major organs (from left to right are heart, liver, spleen, lungs and kidneys, respectively) at 72 h after administration. (D) Representative images of F4/80 (green channel) positive hepatic macrophages incorporated FT@XBP1 (red channel) in the liver tissues from MASH mice (scale bar = 20/50 μm), and (E) the fluorescence intensity was measured. The protein (F) and mRNA (G) levels of *Xbp1s* in liver tissues of FT@XBP1 treated MASH mice or relative control. (H) IF analysis XBP1 expression in F4/80 (red channel) positive hepatic macrophages, and (I) the fluorescence intensity was measured. Data are presented as the means ± SD (error bar) of at least three independent experiments. ***P < 0.001 as indicated.

To explore the precise role of FT@XBP1 on macrophages, we firstly determined whether FT@XBP1 could be effectively taken up by macrophages, and found that rhodamine B-labeled FT@XBP1 nanomembranes were successfully swallowed into the cytoplasm and partially entered the nucleus (Fig. 4A). Moreover, macrophages (RAW 264.7 and mTHP-1) co-incubated with either LPS (M1 phenotype) or IL4 + IL-13 (M2 phenotype) could uptake more red fluorescence than untreated macrophages (Fig. 4A). Consistently, FT@XBP1 can be engulfed by RAW 264.7 cells through TEM analysis (Fig. S5). Subsequently, FT@XBP1 was injected intravenously through the tail vein in Kunming mice, and *in vivo* imaging technology was employed to detect the fluorescence intensity. The fluorescence intensity of rhodamine B increased over time and reached its peak at 24 h (Fig. 4B). In addition, FT@XBP1 was mainly enriched in the liver and was not completely metabolized 72 h after injection (Fig. 4B). Therefore, FT@XBP1 was administered every three days. Moreover, the near-infrared imaging of the heart, liver, spleen, lungs, and kidneys also revealed that fluorescence was concentrated in the liver (Fig. 4C). Then, the uptake of FT@XBP1 by macrophages *in vivo* was confirmed using IF assays. Rhodamine B-labeled FT@XBP1 was mainly colocalized with F4/80*^+^* macrophages, indicating that FT@XBP1 was delivered to the liver and taken up by hepatic macrophages (Fig. 4D and Fig. 4E). XBP1s was highly expressed in the liver tissues of mice fed with an FFC diet for 24 weeks compared with normal mice, and the trend was partially reversed by FT@XBP1 nanomaterials both at the protein (Fig. 4F) and mRNA levels (Fig. 4G). More importantly, XBP1 was significantly increased in F4/80*^+^* macrophages in mice fed with an FFC diet for 24 weeks, while FT@XBP1-treated mice demonstrated significantly lower levels of XBP1s (Fig. 4H and Fig. 4I). Collectively, the FA-TPGS system has the potential to deliver *Xbp1* siRNA to hepatic macrophages and then specifically deregulated *Xbp1* in macrophages.

### 3.4 FT@XBP1 alleviated hepatic steatosis, injury, and fibrosis in MASH mice

**Figure 5:**
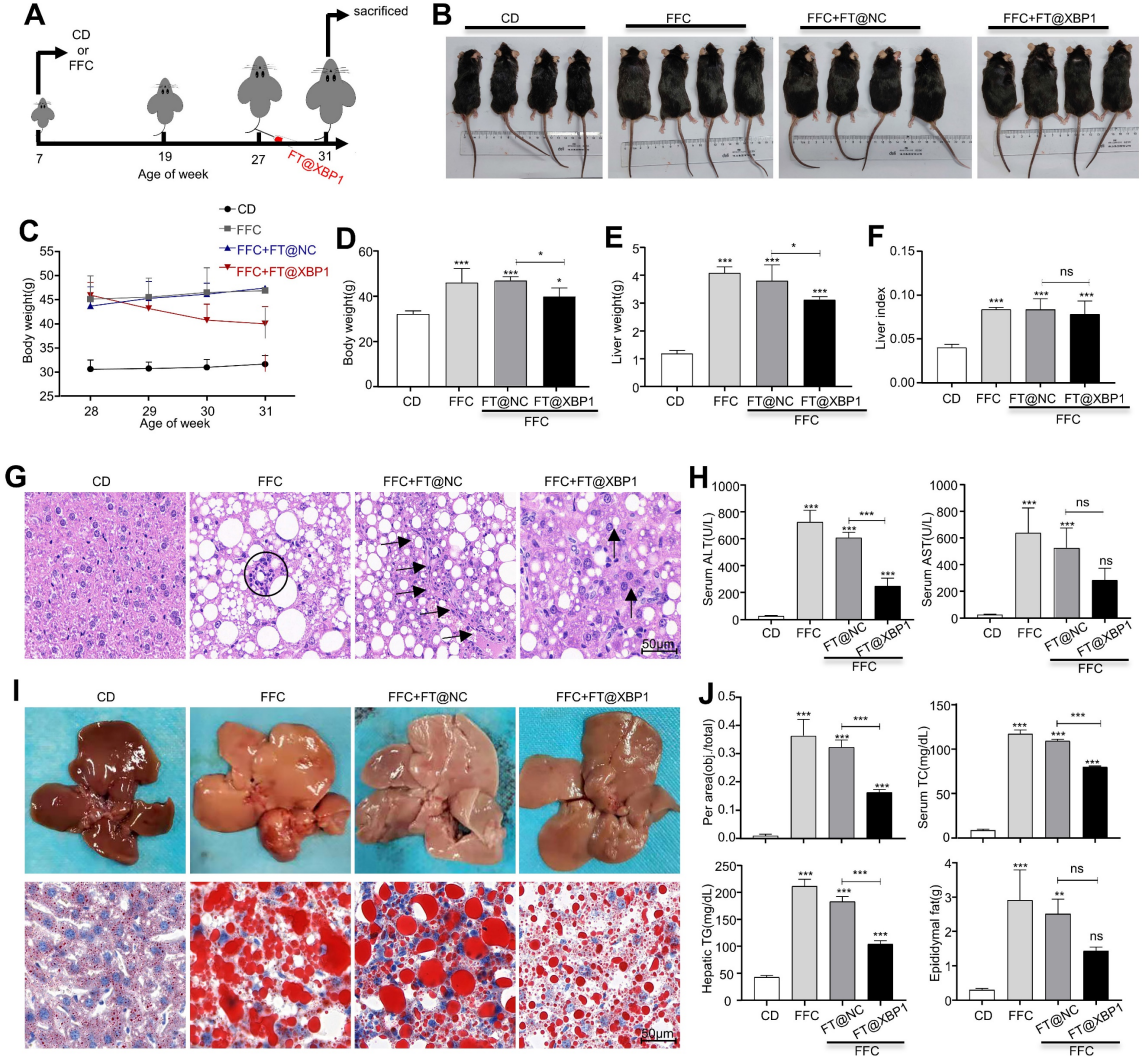
** FT@XBP1 alleviated liver injury and steatosis in FFC dieted MASH mice.** (A) Schematic overview of the strategy for measuring the effect of FT@XBP1 on FFC diet-induced MASH. (B) Images of mice treated with FT@XBP1 or control. (C, D) Body weight, (E) liver weight and (F) liver index of FFC-fed or chow-fed mice, and FFC-fed mice treated with FT@XBP1 or FT@NC (n = 4). (G) Representative images of H&E staining (scale bar = 50 μm). (H) Serum ALT and AST levels. (I) Images of liver tissues and Oil red O staining (scale bar = 50 μm), and (J) the levels of hepatic TG, serum TC and epididymal fat in FT@XBP1 treated MASH mice or control (n = 4). Data are presented as the means ± SD (error bar) of at least three independent experiments. *P < 0.05, **P < 0.01 and ***P < 0.001 as indicated.

The hepatoprotective effect of *Xbp1* deletion in macrophages was further investigated in a MASH mouse model. *Xbp1* was deleted in hepatic macrophages after 20 weeks of FFC feeding by injecting FT@XBP1 intravenously every three days for 4 weeks (Fig. 5A). Mice fed with an FFC diet and FFC mice injected with FT@NC had a higher body weight (Fig. 5B-5D) than mice fed with CD and mice treated with FT@XBP1 (Fig. 5B, 5C, and Fig. 5D). FFC diet feeding also significantly increased the liver weight (Fig. 5E) and the liver-to-weight ratio (Fig. 5F), whereas FT@XBP1 injection remarkably decreased the liver weight, but had no effect on the liver-to-weight ratio compared with FFC diet-fed mice (Fig. 5E and Fig. 5F). Moreover, the effect of FT@XBP1 treatment on hepatic lipid accumulation and injury was evaluated using hematoxylin and eosin staining. An overall favorable effect on steatohepatitis-related parameters was noted in the FT@XBP1 group, including a decrease in steatosis percentage, intrahepatic ballooning, and lobular inflammation, which improved liver histology (Fig. 5G). FFC diet-induced mice had high levels of serum alanine transaminase (ALT) and aspartate aminotransferase (AST) in comparison with mice with the CD diet, and FT@XBP1 remarkably decreased ALT and AST levels (Fig. 5H). Concerning the therapeutic significance, we found that *Xbp1* deficiency largely attenuated FFC diet-induced hepatic steatosis in mice. The liver of FFC diet-fed mice was larger than that of CD-fed mice (upper panel in Fig. 5I). However, these external changes were significantly improved with FT@XBP1 intervention. Moreover, lipid accumulation was reduced (lower panel in Fig. 5I), and the levels of serum total cholesterol (TC), hepatic total triglycerides (TG), and epididymal fat were decreased (Fig. 5J). The NAFLD score was used for the quantitative evaluation of unique MASH-identified lesions^30^. As shown in Table 1, the FFC diet significantly increased NAFLD scores (NAS > 5) compared with CD, whereas FT@XBP1 treatment remarkably reduced NAS compared with mice fed with an FFC diet or treated with FT@NC (*P* = 0.003), as demonstrated by evident reduced lobular inflammation and steatosis. Our results suggested that hepatic macrophages promote the progression of MASH in an XBP1-dependent manner, and the modulation of XBP1 in macrophages may be a useful strategy to prevent MASH progression.

**Figure 6:**
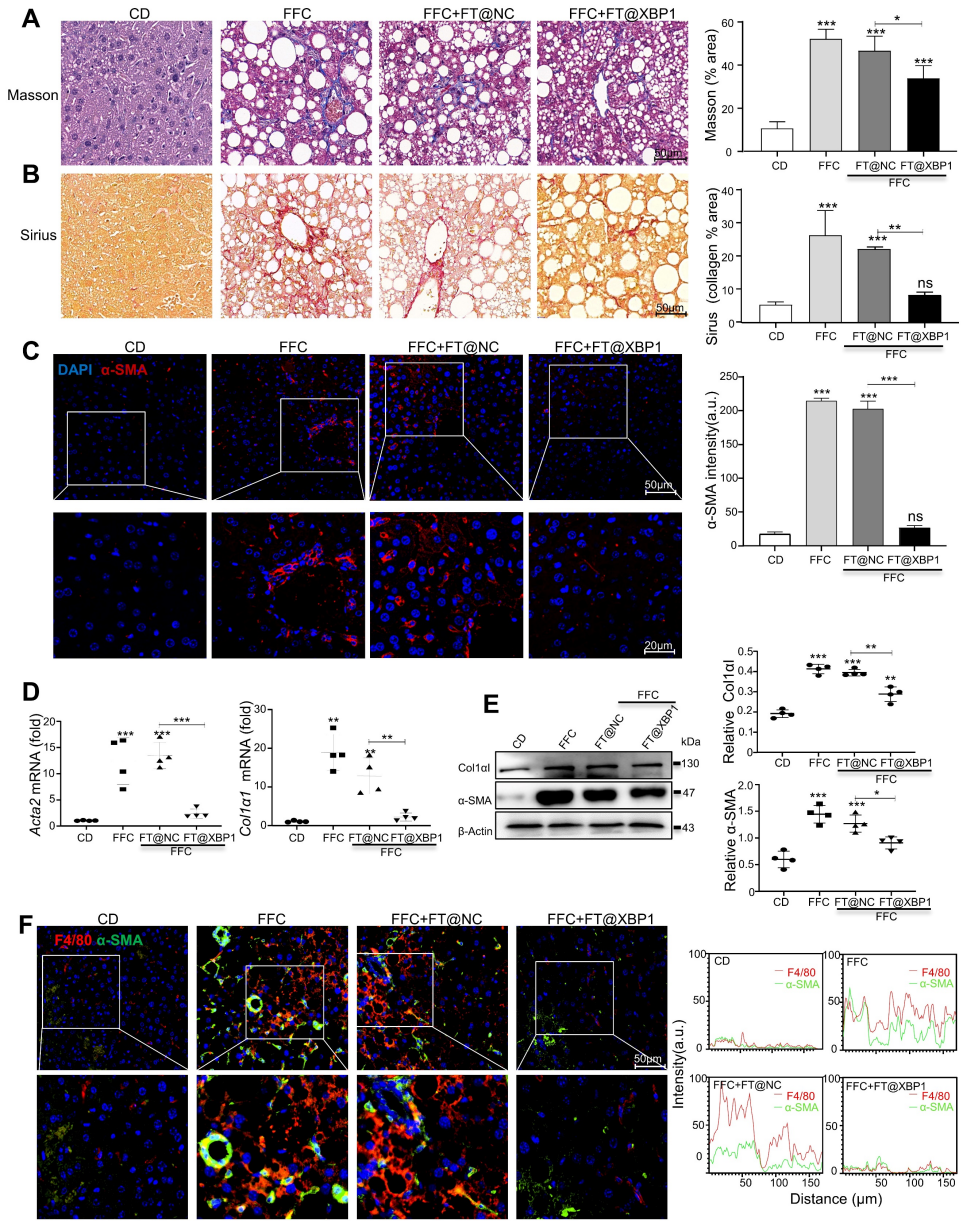
** FT@XBP1 alleviated fibrosis in FFC dieted MASH mice.** The sections of liver tissues from FT@XBP1 treated mice fed with FFC or chow diet were subjected to Masson staining (A) and Sirius red staining (B), and quantitative analyzed (scale bar = 50 μm). (C) Representative immunofluorescence image of α-SMA in MASH livers (scale bar = 50/20 μm), and semi-quantitative analyzed. The expression of fibrotic genes (D) and proteins (E) of α-SMA and col1αI in liver tissues, and band were quantitatively analyzed. (F) Representative immunofluorescence for F4/80 and α-SMA in MASH livers (scale bar = 50/20 μm), and semi-quantitative analyzed. Data are presented as the means ± SD (error bar) of at least three independent experiments. *P < 0.05, **P < 0.01 and ***P < 0.001 as indicated.

Fibrosis is a consequence of chronic liver injury and inflammation and a risk factor for the deterioration of MASH. Therefore, collagen deposition was determined using picrosirius red and Masson staining. The livers of mice fed with an FFC diet demonstrated an evident increase in collagen deposition and pericellular fibrosis compared with CD-fed mice, which were named NASH-associated fibrosis (≥ S2). Nevertheless, these pathological changes were significantly reduced in mice treated with FT@XBP1 (Fig. 6A and Fig. 6B, and Table 1). These results were further confirmed using an IF assay. The expression of profibrogenic protein α-SMA in liver tissues of FT@XBP1-treated mice was reduced compared with the FFC or FT@NC group (Fig. 6C). Indicators of hepatic fibrosis, such as Col1αI and α-SMA, were significantly reduced in mice treated with FT@XBP1 compared with FFC diet-fed mice at both mRNA (Fig. 6D) and protein levels (Fig. 6E). We stimulated mouse HSCs JS-1 with FT@XBP1 nanomaterials *in vitro*, however, FT@XBP1 could not further activate HSCs stimulated with TGF-β1 as demonstrated using Western blot analysis (Fig. S6A), whereas oil red O staining demonstrated that FT@XBP1 slightly decreased lipid accumulation in PA-stimulated AML-12 cells (Fig. S6B). These results suggested that FT@XBP1 treatment ameliorated steatohepatitis and fibrosis in response to FFC diet-induced MASH and FT@XBP1 prevented MASH progression via indirectly targeting HSCs and hepatocytes. Macrophages infiltration and HSCs activation are closely implicated in the progression of MASH. Most interestingly, we found that F4/80^+^ macrophages and α-SMA-positive HSCs were remarkably colocalized in the lesion area, and FT@XBP1 reduced the co-localization (Fig. 6F). Similarly, IHC staining also showed that the number of F4/80^+^ macrophages and α-SMA^+^ HSCs significantly increased in the livers of FFC diet-fed mice (Fig. S7A and Fig. S7B). These results suggested that the fibrosis reduction took place secondary to the loss of hepatic macrophage XBP1 signaling with improvements in liver injury and inflammation.

### 3.5 FT@XBP1 transformed macrophages into the M2 phenotype

**Figure 7:**
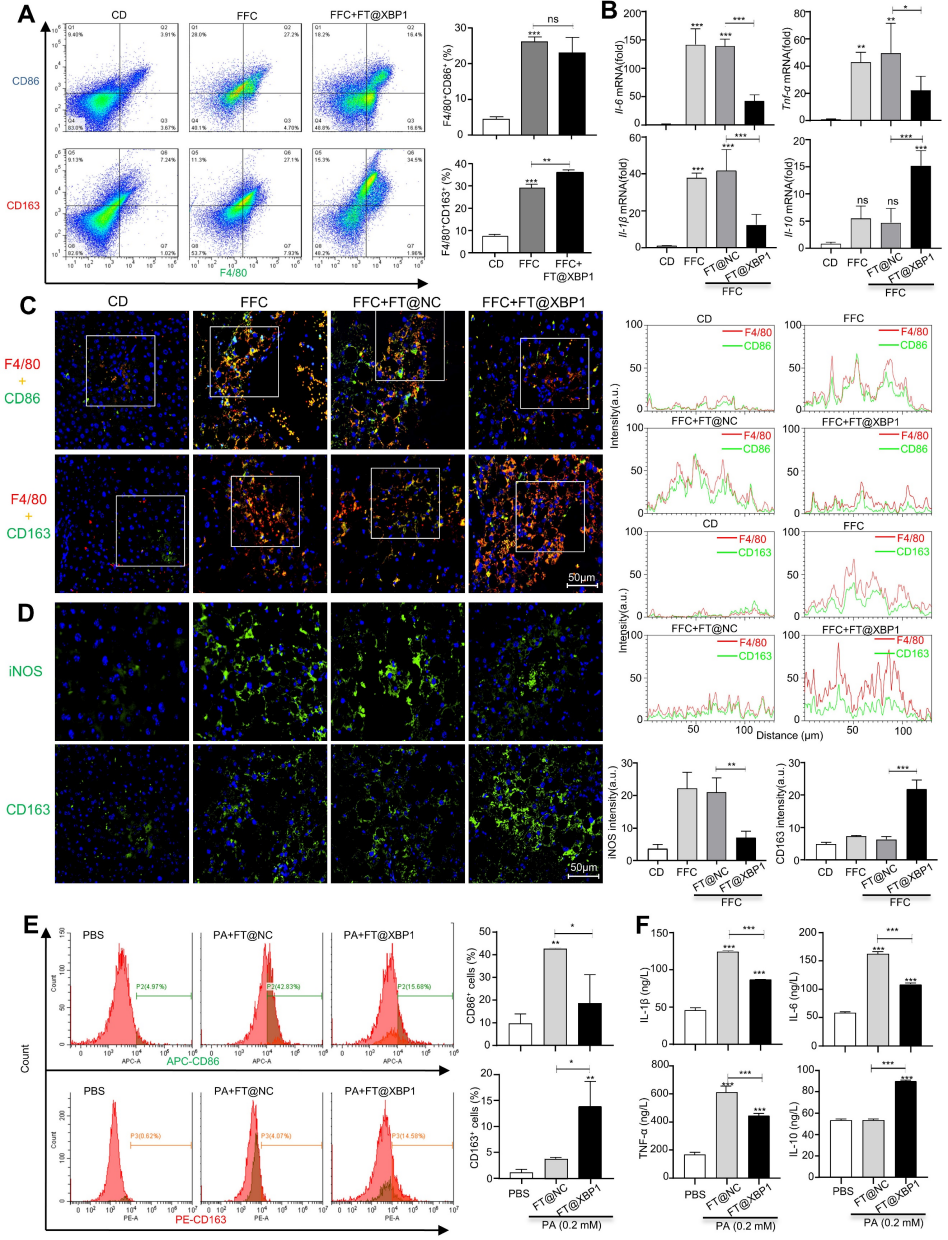
** FT@XBP1 repolarized M1 macrophages toward M2 phenotype *in vitro* and *in vivo*.** (A) Flow cytometry detected the phenotypes of hepatic macrophages from FT@XBP1 treated mice. (B) Inflammatory factors *Il-6, l-1β, Tnf-*α and *Il-10* in hepaticliver tissues of FT@XBP1 treated mice or relative controls were measured using qRT-PCR. (C) Representative immunofluorescence for dual staining F4/80 and CD86 or F4/80 and CD163 in the livers of FT@XBP1 treated mice or relative controls, and semi-quantitative analyzed (scale bar = 50 μm). (D) Representative images of iNOS and CD163 in livers of MASH mice, and quantitative analyzed (scale bar = 50 μm). (E) Flow cytometry detected the percentage of CD86^+^ and CD163^+^ RAW 264.7 cells after co-incubating with PA and/or FT@XBP1, and (F) the cultured supernatants were used for measuring inflammatory factors IL-6, IL-1β, TNF-α and IL-10. Data are presented as the means ± SD (error bar) of at least three independent experiments. *P < 0.05, **P < 0.01 and ***P < 0.001 as indicated.

Inflammatory cell infiltration is another major pathological feature of MASH in addition to hepatocellular injury^5^. Hepatic macrophages, including Kupffer cells and infiltrated monocytes, play a crucial role in the development of MASH. Therefore, we measured the effect of FT@XBP1 on hepatic macrophages in FFC diet-induced MASH. F4/80^+^CD86^+^ represented the M1 phenotype and F4/80^+^CD163^+^ indicated the M2 phenotype in mouse liver. In comparison with normal CD mice, FFC diet-fed mice showed a significant increase in F4/80^+^CD86^+^ M1 and F4/80^+^CD163^+^ M2 macrophages (Fig. 7A). FT@XBP1 treatment slightly decreased the proportion of F4/80^+^CD86^+^ cells; however, the F4/80^+^CD163^+^ proportion significantly increased compared with FFC diet-induced MASH mice as measured using flow cytometry analysis (Fig. 7A). In addition, we found that FT@XBP1 treatment significantly reduced the mRNA levels of pro-inflammatory factors (e.g.,* Il-6, Il-1β, and Tnf-*α), but increased the expression of *Il-10* mRNA compared with FFC and FT@NC groups in liver tissues (Fig. 7B). Subsequently, we employed the multilabel IF staining of F4/80, CD163, and CD86 in liver tissues and found that lesions from FFC diet-fed mice had a higher infiltration of F4/80^+^CD86^+^ cells, whereas FT@XBP1 significantly reduced the number of F4/80^+^CD86^+^ cells but increased the number of F4/80^+^CD163^+^ cells (Fig. 7C). This phenomenon was further confirmed in multilabel IF staining of liver tissues of FFC-fed mice with CD11b, CD163, and CD86 (Fig. S8A). Moreover, the IF staining of liver tissue with iNOS (an M1 marker) and CD163 showed that FT@XBP1 treatment reduced the number of iNOS-positive cells, while CD163^+^ cells were significantly increased compared with FFC-fed mice or those treated with FT@NC (Fig. 7D). Together, our results suggested that FT@XBP1 reprogrammed macrophages toward the M2 phenotype in the liver of MASH mice.

It has been found that free fatty acids, for example, PA, can directly activate macrophages^31^. Herein, RAW 264.7 cells were stimulated with 0.2 mM PA for 24 h *in vitro* to mimic the high lipid state in MASH and found that PA-induced RAW 264.7 cells polarize toward the M1 phenotype, which showed the increased expression of CD86 (Fig. S8B), and pro-inflammatory factors, such as IL-6, IL-1β, and TNF-α, but had little effect on IL-10 (Fig. S8C). Moreover, FT@XBP1 neutralized the effects of PA and promoted RAW 264.7 cells phenotype shift from M1 to M2, which could be supported by reduced CD86^+^ cells but increased CD163*^+^* proportion (Fig. 7E). Consistently, FT@XBP1 significantly decreased the expression levels of IL-6, IL-1β, and TNF-α but increased IL-10 levels in RAW 264.7 cells compared to the FT@NC group (Fig. 7F). These results suggested that FT@XBP1 ameliorated MASH by converting pro-inflammatory hepatic macrophages to M2 type macrophages.

### 3.6 FT@XBP1 alleviated MASH through exosome mediated-inhibition of HSCs activation

**Figure 8:**
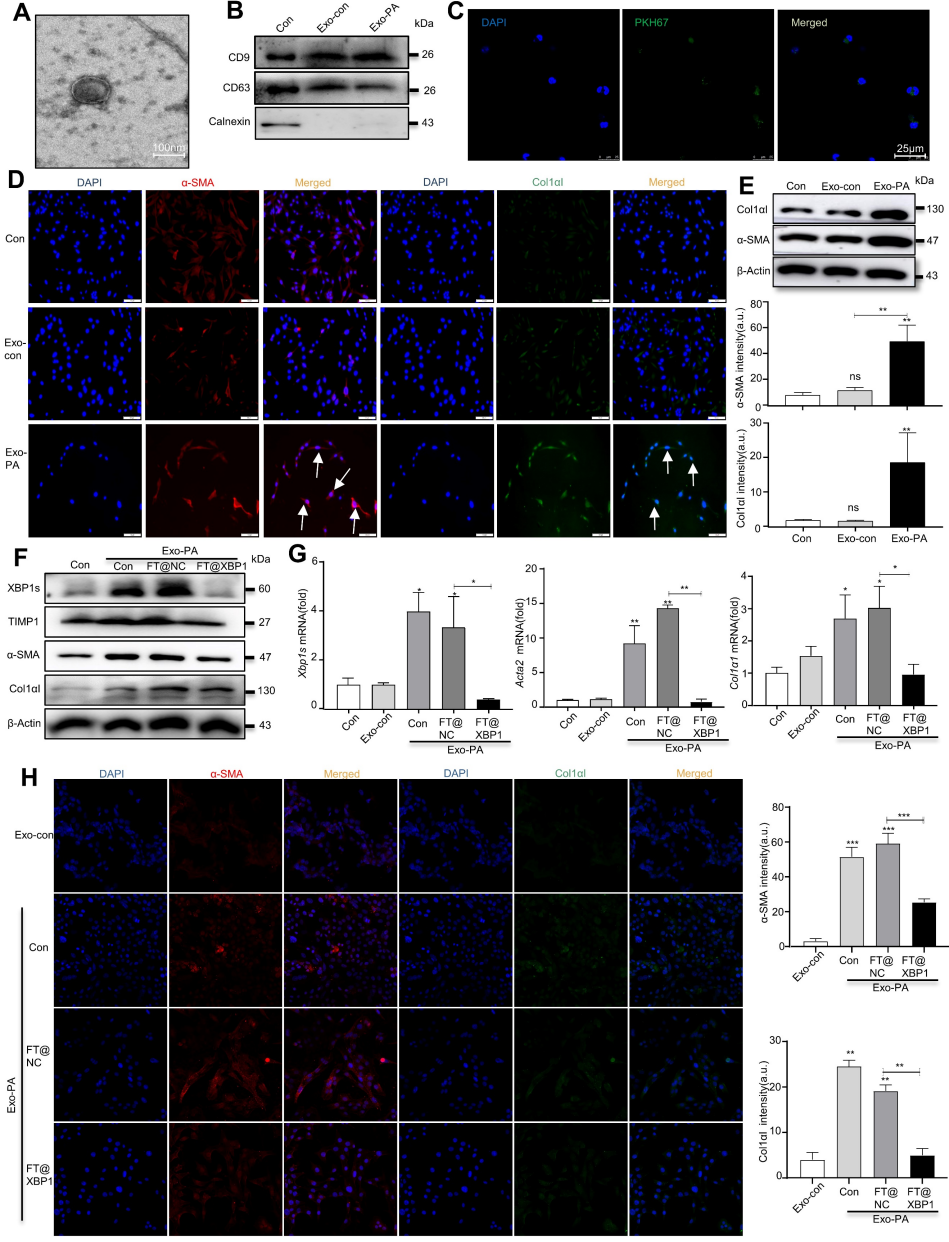
** FT@XBP1 alleviated HSCs activation via macrophages derived exosomes.** (A) TEM image of exosomes derived from PA treated RAW 264.7 cells (scale bar = 100 nm). (B) Western blot analysis of CD9, CD63, and Calnexin using cell lysates or purified exosomes. (C) Representative images of JS-1 cells encapsulated PKH67-labeled exosomes *in vitro* (scale bar = 25 μm). The expression of fibrotic molecules was measured by immunofluorescence staining (D) and Western blot analysis (E) of JS-1 cells stimulated with Exo-PA or Exo-con, scale bar = 50 μm. The expression of XBP1s, α-SMA, TIMP1 and Col1αI was determined by Western blot analysis (F), and qRT-PCR assay (G) in JS-1 cells. (H) Immunofluorescence staining of α-SMA and Col1αI after JS-1 cells were treated with macrophage-derived exosomes *in vitro* (scale bar = 25 μm). Data are presented as the means ± SD (error bar) of at least three independent experiments. *P < 0.05, **P < 0.01 and ***P < 0.001 as indicated.

Exosomes can encapsulate biological active substances such as genetic material, lipids, and proteins of parent cells and deliver them to recipient cells in the microenvironment to directly or indirectly regulate the biological behaviors of recipient cells. We previously found that ER-stressed hepatocellular carcinoma cells modulate the function of macrophages by transmitting miRNA-containing exosomes^32^; thus, we wondered whether high-fat-induced ER-stressed hepatic macrophages modulate MASH progression via exosomes. To confirm this hypothesis, we purified exosomes from the supernatants of PA- and/or FT@XBP1-treated RAW 264.7 cells or paired control using the ExoQuick precipitation solution. TEM was used to identify whether the precipitated vesicles had the typical morphology of exosomes. Precipitated plates demonstrated round or round-like bilayer vesicles (Fig. 8A), which was consistent with the morphological features of exosomes. Several exosomal biomarkers were detected using Western blot analysis to confirm whether the precipitate vesicles were exosomes. These round vesicles were positive for endosomal-specific transmembrane proteins CD9 and CD63, while negative for the ER protein calnexin (Fig. 8B). Moreover, cocultured JS-1 cells with PKH67-labeled exosomes for 12 h showed green fluorophore under confocal microscope (Fig. 8C), suggesting that the exosomes could be effectively incorporated and then mediated intercellular communication.

Previous studies have demonstrated that PA can promote hepatocyte IRE1α activation and release exosomes to upregulate ceramide *de novo* synthesis, which improves the inflammatory response in MASH mice^18^. To explore whether exosomes are involved in the “cross-talk” between hepatic macrophages and HSCs activation, we stimulated JS-1 cells with PA directly and found that the expression level of α-SMA did not change compared with the control group (Fig. S9A). However, exosomes derived from PA-treated RAW 264.7 cells (hereafter named Exo-PA) increased the expression levels of α-SMA and Col1αI in JS-1 cells as demonstrated using the IF assay (Fig. 8D). Western blot analysis further confirmed that Exo-PA significantly increased the expression levels of α-SMA and Col1αI (Fig. 8E). Collectively, these results suggested that lipotoxicity-induced macrophage ER stress may mediate the activation of HSCs in an XBP1s-exosome dependent manner.

The effects of exosomes derived from FT@XBP1 transfected macrophages, which were pretreated with PA (hereafter named Exo-FT@XBP1), on the activation of JS-1 cells were examined. Exo-PA significantly increased the expression of XBP1s and fibrosis metrics in JS-1, while Exo-FT@XBP1 showed an opposite effect on the protein expressions of XBP1s, α-SMA, TIMP1, and CoL1αI (Fig. 8F and Fig. S9B) and the mRNA levels of* Xbp1s*, *Acta2,* and *Col1α1* in JS-1 (Fig. 8G). Consistently, images captured using confocal microscope also showed that Exo-FT@XBP1 significantly inhibited the expressions of α-SMA and Col1αI compared with Exo-FT@NC and Exo-PA in JS-1 cells (Fig. 8H). Taken together, our results suggested that ER-stressed hepatic macrophages inhibit the activation of HSCs via an XBP1s-mediated exosome pathway.

## 3. Discussion

This study aimed to decipher how ER-stressed macrophages interacted with HSCs to promote MASH progression, which provides a rational basis for formulating effective treatment strategies. Herein, we illustrate that XBP1s plays a pivotal role in MASH progression using FA-modified TPGS nanoparticles encapsulated with *Xbp1*-specific siRNA (FT@XBP1). Under lipotoxic conditions, hepatic macrophages displayed pro-inflammatory and profibrotic profiles along with significant upregulation of the IRE1α-XBP1 pathway both *in vitro* and *in vivo* and exacerbated MASH progression. FT@XBP1 can be effectively incorporated by hepatic macrophages, and treatments with FT@XBP1 reduced the expression of XBP1s both in primary and established macrophages as well as in mouse liver tissues. Furthermore, hepatic macrophage-specific deletion of *Xbp1* in mice alleviated liver injury, which was manifested by decreased tissue inflammation, recruitment of CD11b positive macrophages, and progressive formation of hepatic fibrosis. Mechanically, lipotoxic stress reprogrammed macrophages and their interaction with HSCs to protect against MASH development in an XBP1s-exosome-dependent manner. These results, coupled with the observation that specifically deleting *Xbp1* in hepatic macrophages can prevent MASH induced by FFC diet feeding, provide strong experimental evidence for FT@XBP1 as an attractive treatment strategy for MASH progression.

Macrophages can modulate the progression of MASH according to their phenotypes^33^. However, the exact role of macrophage plasticity in regulating MASH development still needs to be fully illustrated. Herein, we found that the FFC diet can activate macrophages, which exhibit a hybrid M1/M2 phenotype as demonstrated by increasing levels of both M1 and M2 phenotype-associated biomarkers. It has been proposed that lipids can induce M1 and M2 heterozygous macrophage phenotypes in pro-inflammatory and anti-inflammatory independent pathways^34^ and is considered a metabolically activated macrophage phenotype in a recent review^35^. Moreover, several studies have suggested that ER stress may be a necessary factor for macrophages to generate M2 phenotype transformation^36^. In addition, the effects of XBP1 signaling on macrophage activation have been discussed recently^22^, ^37^. In the current study, we elucidated that pro-inflammatory hepatic macrophages are players orchestrating hepatic inflammation and HSCs activation, both of which promote MASH progression and the ER stress key transcription factor (XBP1s) underlies macrophage regulation of hepatic steatosis, inflammation, and fibrosis. Therefore, regulating XBP1s to restore ER homeostasis in macrophages may represent a promising strategy for the prevention and treatment of MASH.

ER stress has been observed in nearly all chronic liver diseases and is distinguished by the activation of three UPR pathways^38^. IRE1α-XBP1 is the most conservative pathway in the UPR response, and activation of the IRE1α-XBP1 pathway can arise under both proteotoxic- and lipotoxic conditions^39^, ^40^. Consistent with the latter, herein, we found that the IRE1α-XBP1 pathway is activated in macrophages both in a dietary MASH mouse model and PA-induced lipotoxic stress *in vitro*. Several studies have indicated that the IRE1α-XBP1 pathway can alleviate ER stress-associated hepatic steatosis in mice^41^; however, IRE1α was confirmed to increase lipid accumulation in liver IRE1α specifically knockout mice^42^. In addition, independent investigators have contradictorily reported that XBP1 may serve as a pro-lipogenic or anti-lipogenic gene dependent on animal models with genetic- or adenovirus-based ablation of XBP1 in the liver^43^, ^44^. Our *in vitro* and *in vivo* results supported that lipotoxic stress in macrophages promoted MASH progression in an XBP1-dependent manner, highlighting a promising strategy for MASH macrophages.

Effective treatment of MASH via targeting ER-stressed macrophages remains a great challenge due to the lack of macrophage-specific drug delivery methods. TPGS is a hydrophilic derivative of natural vitamin E, which contains both lipophilic and hydrophilic components of polyethylene glycol^45^. TPGS has increased drug loading and entrapment efficiency, more than 67 times compared with polyvinyl alcohol^46^ and can enhance the encapsulation efficiency and bioavailability of certain hydrophobic drugs through different biological barriers^47^. Therefore, TPGS may be a preferred choice to facilitate the formation of siRNA-based treatment strategy. There are four types of FRs, including FRα, FRβ, FRγ and FRδ. Of those, FRα is expressed on normal epithelial cells (e.g. kidney, spleen and lung tissue) and tumor tissues^48^, while FRβ is selectively expressed on cells of the myeloid lineage and is upregulated on activated macrophages^49^. More importantly, FRα and FRβhave demonstrated high affinity and low immunogenicity. Herein, we successfully coupled FA with TPGS and generated *Xbp1* siRNA encapsulated FT@XBP1 nanomaterials. These FT@XBP1 nanocarriers were specifically engulfed by hepatic macrophages both *in vitro* and* in vivo* through receptor-mediated endocytosis, which is consistent with other studies^50^.

In addition, we noticed that these *Xbp1* siRNA encapsulated FA-TPGS nanocarriers significantly reduced the level of XBP1 in macrophages and promoted macrophages to transform to an anti-inflammatory phenotype under lipotoxic stress. Moreover, FT@XBP1 alleviated hepatic steatosis, injury, and fibrosis in an FFC diet-induced MASH model, while the impact of FT@XBP1 on HSCs *in vitro* was almost negligible. We discovered that FT@XBP1 reprogrammed macrophages from the M1 to the M2 phenotype and increased exosomes released by M2 macrophages, which further inhibited the activation of HSCs. Collectively, our findings support the idea that FT@XBP1 can specifically target hepatic macrophages and alleviate MASH by repolarizing macrophages.

## 4. Conclusions

In summary, this study constructed folate-modified TPGS nanocarriers, which could effectively encapsulate *Xbp1* siRNA and be specifically engulfed by hepatic macrophages *in vitro* and *in vivo*. Moreover, FT@XBP1 had the following functions: reducing the expression of XBP1s, reprogramming macrophages toward the M2 phenotype under lipotoxic stress conditions, and subsequently ameliorating liver injury, infiltration of inflammatory cells, collagen deposition, and fibrosis in the FFC diet-induced MASH model. Our innovative study reveals the great potential of folate-modified TPGS nanocarriers and paves the way for the therapeutic intervention of MASH patients.

## Supplementary Material

Supplementary materials and methods, tables, figures.

## Figures and Tables

**Table 1:** NAFLD activity score of mice treated with FT@XBP1

	CD	FFC	FFC + FT@NC	FFC + FT@XBP1
NAS (Mean ± SD)	2.25 ± 0.41	10.25 ± 0.41	11 ± 0.35	6.5 ± 0.75
Steatosis grade (1/ 2/ 3)	1/ 1/ 1/ 1	3/ 3/ 3/ 3	3/ 3/ 3/ 3	2/ 1/ 1/ 2
Lobular inflammation (0/ 1/ 2/ 3/ 4)	0/ 0/ 0/ 0	3/ 4/ 4/ 3	4/ 3/ 4/ 4	3/ 2/ 2/ 3
Fibrosis stage (0/ 1/ 2/ 3/ 4)	1/ 0/ 1/ 1	2/ 2/ 2/ 1	2/ 2/ 3/ 2	2/ 1/ 1/ 1
Ballooning (0/ 1/ 2)	1/ 0/ 0/ 1	2/ 2/ 2/ 2	2/ 2/ 2/ 2	1/ 1/ 1/ 2
