# Supplementary material for: siRNA-loaded folic acid-modified TPGS alleviate MASH via targeting ER stress sensor XBP1 and reprogramming macrophages
Source: Int J Biol Sci. 2024 Jul 8;20(10):3823–41. doi: 10.7150/ijbs.96113 (PMC11302883; doi:10.7150/ijbs.96113)
Supplement: Supplementary file 1 — Supplementary materials and methods, tables, figures. [file ijbsv20p3823s1.pdf]

Supporting Materials

siRNA-loaded folic acid-modified TPGS alleviate MASH *via* targeting ER stress sensor XBP1 and reprogramming macrophages

Manman Zhu[1,2#], Yong Cheng[1,2#], Li Zuo[3], Bao Bin[4], Haiyuan Shen[1,5], Tao Meng[6], Zihao Wu[7], Peng Rao[1,2], Yue Tang[1,2], Shuojiao Li[8], Honghai Xu[7], Guoping Sun[5], Hua Wang[5*], Guiyang Zhang[6*], Jiatao Liu[2*]

[1]School of Pharmacy, Anhui Medical University, Hefei 230032, Anhui Province, China

[2]Department of Pharmacy, the First Affiliated Hospital of Anhui Medical University, Hefei 230022, Anhui Province, China

[3]Department of Pathology, Laboratory of Mucosal Barrier Pathobiology, Anhui Medical University, Hefei 230032, Anhui, China.

[4]Boston Children's Hospital, Harvard Medical School

[5]Department of Oncology, the First Affiliated Hospital of Anhui Medical University, Hefei 230022, Anhui Province, China

[6]Department of Pharmacology, School of Basic Medical Sciences, Anhui Medical University, Hefei 230032, Anhui Province, China

[7]Department of Pathology, The First Affiliated Hospital of Anhui Medical University, Hefei 230022, Anhui Province, China

[8]Department of Pharmacy, Anhui University of Chinese Medicine, Hefei 230012, Anhui Province, China

[#]These authors contributed equally to this work

Contents of the Supporting Data

Contents	Page
Supporting Materials and Methods	Page 2-Page 5
Supporting Tables	Page 6-Page 7
Supporting Figure Legends	Page 8-Page 17

Supporting Materials and Methods

Cell culture

Murine hepatic stellate cell lines JS-1 and murine macrophages cell lines RAW 264.7 were both purchased from Procell company (Wuhan, China) and cultured in the Dulbecco's modified Eagle's medium (DMEM; Hyclone, China) supplemented with 10% fetal bovine serum (FBS, Gibco, USA) and 1% penicillin/streptomycin solution (Beyotime, Shanghai, China) at 37°C in a humidified 5% CO2 atmosphere. Human acute monocytic leukemia cell THP-1 was obtained from Chinese Academy of Sciences (Shanghai, China) and cultured in Roswell Park Memorial Institute (RPMI-1640; Hyclone, China) medium containing 10% FBS (Gibico, USA). In all experiments, THP-1 cells were treated with 100nM phorbol12-myristate13-acetate (PMA, Sigma, P8139) for 24 h to transform into adherent macrophages (mTHP-1). The alpha mouse liver-12 (AML12, ATCC CRL-2254) cells were kindly gifted from Wang Hua's group at Anhui Medical University and cultured in Dulbecco's Modified Eagle Medium (DMEM)/Nutrient Mixture F-12 (1:1) supplemented with 10 % (*v*/*v*) FBS (Gibco, USA).

Reagents and antibodies

Rhodamine B isothiocyanate obtained from Sigma-Aldrich Biological company (MO, USA, Cas#36877-69-7, MW: 536.08). Vitamin E-TPGS was from Macklin Chemical Reagent company (Shanghai, China). Folic acid (FA), Cinnamyl-3,4-dihydroxy-α-cyanocinnamate(CDC), p-toluenesulfonyl chloride (PTSC),

dichloromethane (CH_2Cl_2), N, N-dimethylformamide, triethylamine and 1,4 dioxane were all purchased from Aladdin Chemical Reagent company (Shanghai, China). $NH_2CH_2CH_2NH_2$ was obtained from Sigma-Aldrich Biological company (MO, USA). DAPI with anti-fluorescence quencher and PKH67 Green Fluorescent Cell Linker Mini Kit purchased from Sigma-Aldrich Biological company (MO, USA). Cell Counting kit-8 were obtained from BD Biosciences (Mississauga, CA). Lipopolysaccharide (LPS)，IL-4，IL-13 and phorbol myristate acetate (PMA) was from Sigma-Aldrich Biological company (MO, USA). TRIzol reagent, First Strand cDNA Synthesis Kit，BCA kit and qPCR Detection Kit were bought from Thermo Fisher Scientific Co., Ltd (MA, USA). Detailed information about antibodies used in this study was list in **Supporting Table 1**.

Screening *XBP1* siRNA sequences

Three small interfering RNA sequences of *XBP1* (si*XBP1*) and non-silencing RNA (si*NC*) were all obtained from Geenpharma company (Shanghai, China), details are depicted in **Supporting Table 2**. For screening specific si*XBP1,* RAW 264.7 cells were plated at density of 1.5×10^5 cells/mL in DMEM medium without antibiotic, and were transfected with si*NC* and si*XBP1* in antibiotic-free opti-MEM medium (Gibco, USA) using Advanced DNA RNA transfection reagent (Zeta-life, USA) for 48h. Then, cells were collected and the protein levels of XBP1s were detected using western-blotting analysis.

Transmission electron microscope

RAW 264.7 cells were placed in 6-well plates (5×10^5 cells/well), and were transfected with FA-TPGS or FT@XBP1 nano-membranes for 24h. Briefly, sample were prepared as previously reported[1] and images were captured at 80KV using a Talos L120C G2 (Thermo, USA) transmission electron microscope.

Induction of M1/M2 macrophages *in vitro*

RAW 264.7 or mTHP-1 cells were seeded in plates (5×10^5 cells/well). Lipopolysaccharide (LPS,100 ng/mL) or IL-4 (20ng/mL) and IL-13 (20ng/mL) were co-cultured with RAW 264.7 and mTHP-1 cells for 24h to differently establish M1 or M2 phenotype macrophages, respectively.

Induction of ER stress *in vitro*

A series concentration (0, 0.3125, 0.25, 1.25, 2.5 and 5.0μM) of tunicamycin (Solarbio, IT2670, China) was used to stimulate RAW 264.7 and mTHP-1 cells and induce endoplasmic reticulum (ER) stress. To construct a high-fat induced ER stress model in macrophages, a body of concentrations of palmitic acid (PA, 0, 0.1 and 0.2mM) and 10% fatty acids-free bovine serum albumin (BSA, Sigma, SRE0098) were well mixed (molar ratio PA and BSA 3:1) and co-cultured with RAW 264.7 and mTHP-1 cells for 24 hours. For measuring the role of 4μ8C (Selleck, S7272, USA) on the expression of XBP1s, concentration of 4μ8C (0, 20, 40, 60, 80 and 100μm) was employed to stimulate RAW 264.7 cells 24 hours.

The impact of FT@XBP1 on JS-1 activation

Mouse hepatic stellate cells (JS-1) were inoculated in 6-well plates for 12h at a density of 3×10^5 cells/well and switched to serum-free and antibodies-free DMEM medium for 12h. JS-1 cells pre-treated with 10ng/mL TGF-β1 (Peprotech, USA) for 24h to transform into activated hepatic stellate cells. And then FA-TPGS, FT@NC, FT@XBP1 nanocarriers were applied to stimulate JS-1 cells for 48h, qRT-PCR and western-blotting analysis were used to detect the impact of FT@XBP1 on the activation of hepatic stellate cell.

Relative releasing of FT@XBP1

Formula for calculating accumulative drug release.

$$E_r = \frac{V_e \sum_{1}^{n-1} C_i + V_0 C_n}{m_{drug}}$$

E_r: cumulative drug release; V_e: volume of PBS replacement; V_0: total volume of release medium; C_i: concentration of release solution at the i-th replacement; m_{drug}: total mass of drug contained in the nanoparticles; n: number of PBS replacements.

The experiment was repeated three times and the results were averaged with a correlation of $P<0.05$. The cumulative amount of drug released at each sampling point was calculated according to the above equation.

Hepatocyte oil red staining

Oil red o staining was performed in mouse hepatocytes cell lines AML-12 and sections of liver tissues as manufacturer’s instructions (C0158M, Beyotime). Briefly, the cells planted in 6-well plates were stimulated with PA for 24 hours as a positive control. And AML-12 cells were pre-transfected with FT@XBP1 or FT@NC nano-membranes (*c*(FA-TPGS): 2.4μg/mL) for 48 hours and then treated with PA/DMEM for 24 hours. Later, cells were stained with oil-red-o staining solution for 30 mins and images viewed under brightfield by fluorescent microscope (Leica, German).

Reference

[1] J.T. Liu, W.C. Li, S. Gao, F. Wang, X.Q. Li, H.Q. Yu, L.L. Fan, W. Wei, H. Wang, G.P. Sun, Autophagy Inhibition Overcomes the Antagonistic Effect Between Gefitinib and Cisplatin in Epidermal Growth Factor Receptor Mutant Non--Small-Cell Lung Cancer Cells, Clin Lung Cancer 16(5) (2015) e55-66.

Supporting Tables

Table S1 Antibodies used in this study

Antibody	Source	Application
anti-mouse β-actin	Zhongshan	WB
anti-rabbit XBP1s	Cell signal	WB
ant-rabbit XBP1®488	Santa Cruz	IF
anti-rabbit PERK	Cell signal	WB
anti-rabbit IRE1α	Cell signal	WB
anti-rabbit GRP78	Cell signal	WB
anti-rabbit ATF6	Cell signal	WB
anti-mouse α-SMA	ZENBIO	WB、IF
anti-rabbit α-SMA	Bioss	IHC
anti-rabbit Collagen I α1	Bioss	WB、IF
anti-mouse F4/80	Santa Cruz	IF
anti-rabbit Timp1	Abcam	WB
anti-mouse F4/80 (FITC)	Biolegend	FCM
anti-mouse CD86(APC)	Biolegend	FCM
anti-mouse CD163(PE)	Biolegend	FCM
anti-mouse CD63	Abcam	WB
anti-mouse Calnexin	Abcam	WB
anti-mouse CD9	Abcam	WB
anti-rabbit CD86	Bioss	IF
anti-rabbit CD163	Bioss	IF
anti-mouse CD11b	Santa Cruz	IF
anti-rabbit iNOS	Abcam	IF
anti-human CD68	Abcam	IF
anti-human XBP1	Cell signal	IF
Horseradishperoxidase labeled anti-mouse/anti-rabbit IgG	Zhongshan	WB

Supporting table S2 RNA sequence used in this paper

RNA	Sequence	
Mouse XBP1s siNC	Forwardprimer:	UUCUCCGAACGUGUCACGUTT
	Reverseprimer:	ACGUGACACGUUCGGAGAATT
Mouse siXBP1s-1	Forwardprimer:	CUGCUCGAGAUAGAAAGAATT
	Reverseprimer:	UUCUUUCUAUCUCGAGCAGTT
Mouse siXBP1s-2	Forwardprimer:	CAAGCUGGAAGCCAUUAAUTT
	Reverseprimer:	AUUAAUGGCUUCCAGCUUGTT
Mouse siXBP1s-3	Forwardprimer:	CAGAUUCUGAGUCUGAUAUTT
	Reverseprimer:	AUAUCAGACUCAGAAUCUGTT
Mouse Acta2	Forwardprimer:	TGACAGGATGCAGAAGGAGAT
NM_007392.3	Reverseprimer:	TCACAGTTGTGTGCTAGAGGC
Mouse Col1α1	Forwardprimer:	CGACCTCAAGATGTGCCACT
NM_007742.4	Reverseprimer:	CCATCGGTCATGCTCTCTCC
Mouse XBP1	Forwardprimer:	CTGAGTCCGCAGCAGGTG
NM_001271730.1	Reverseprimer:	TTCCAGCTTGGCTGATGAGG
Mouse IL-6	Forwardprimer:	CAACGATGATGCACTTGCAGA
NM_001314054.1	Reverseprimer:	TGTGACTCCAGCTTATCTCTTGG
Mouse IL-1β	Forwardprimer:	TGCCACCTTTTGACAGTGATG
NM_008361.4	Reverseprimer:	TGATGTGCTGCTGCGAGATT
Mouse TNF-α	Forwardprimer:	ACCCTCACACTCACAAACCAC
NM_001278601.1	Reverseprimer:	ATAGCAAATCGGCTGACGGT
Mouse IL-10	Forwardprimer:	TGCAGTGTGTATTGAGTCTGCT
NM_010548.2	Reverseprimer:	GCTCTGTCTAGGTCCTGGAG

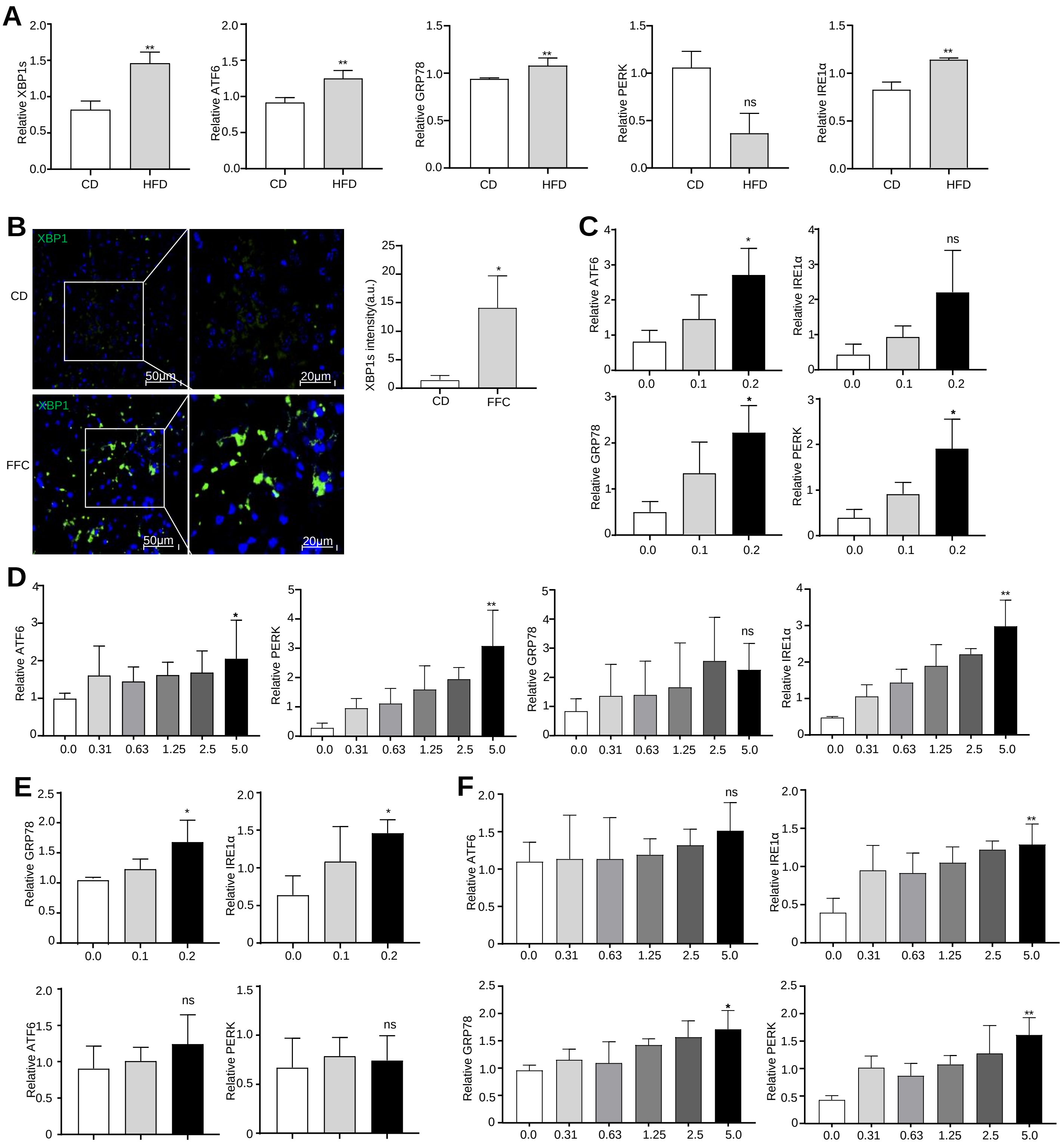

Figure S1. ER stress is activated in high-fat induced NASH model. Semi-quantitatively analyzed the band intensity of ATF6, IRE1α, GRP78 and PERK in Fig.1A (A). (B) Representative immunofluorescence images of XBP1 in FFC diet-induced NASH mice livers (scale bar=50/20μm), and semi-quantitative analyzed. Semi-quantitatively analyzed the band intensity of ATF6, IRE1α, GRP78 and PERK in the left panel of Fig.1D (C), the right panel of Fig.1D (D), the left panel of Fig.1E (E) and the right panel of Fig.1E (F). Data are presented as the means ± SD (error bar) of at least three independent experiments. **P*< 0.05 and **P<0.01 as indicated.

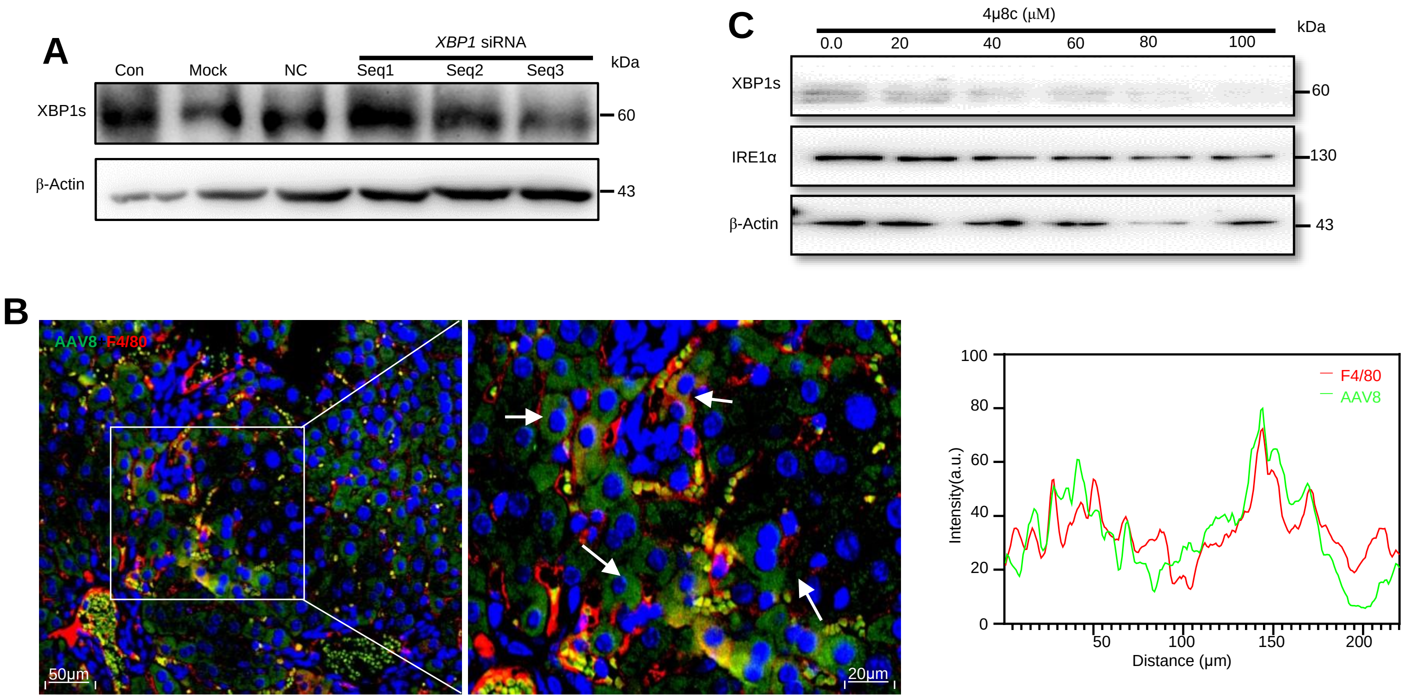

Figure S2. Adeno-associated virus-8 (AAV-8) could not specifically target hepatic macrophages. (A) Western-blotting analyzed the expression of XBP1s in RAW 264.7 cells transfected with specific *XBP1* small interference RNA (siRNA). (B) The representative images of hepatic macrophages (red) uptake siXBP1-contained AAV-8 (green), scale bar=50μm. (C) Western-blotting analyzed the expression of IRE1α and XBP1s in RAW 264.7 cells after co-cultured with a series of 4μ8c.

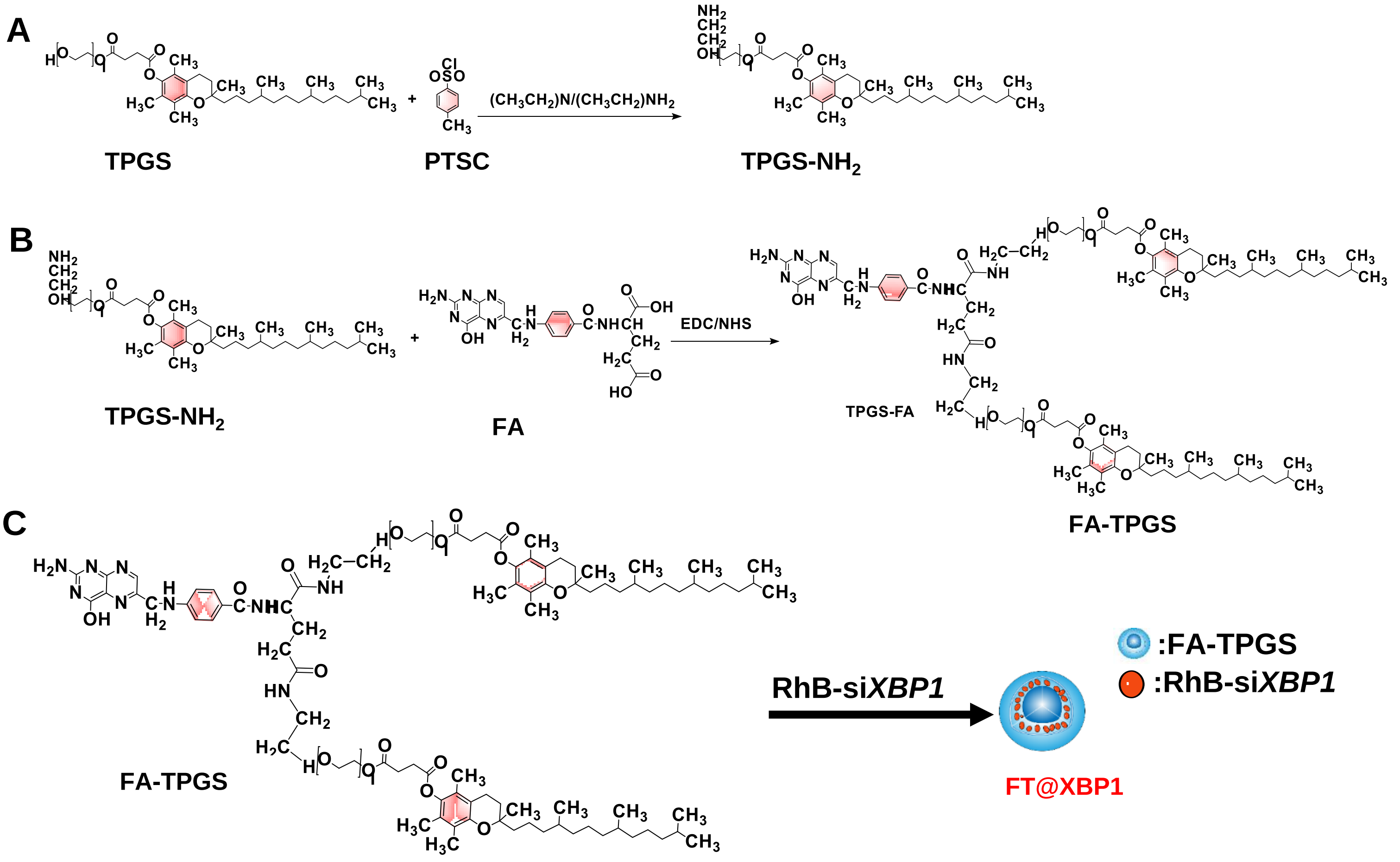

Figure S3. Synthesis of folate-modified TPGS encapsulated with *XBP1* siRNA. (A) The preparation of TPGS-NH2 segments. (B) The synthesis process of FA-TPGS.(C) The formation of *XBP1*-siRNA encapsulated FA-TPGS (FT@XBP1) or relative control (FT@NC).

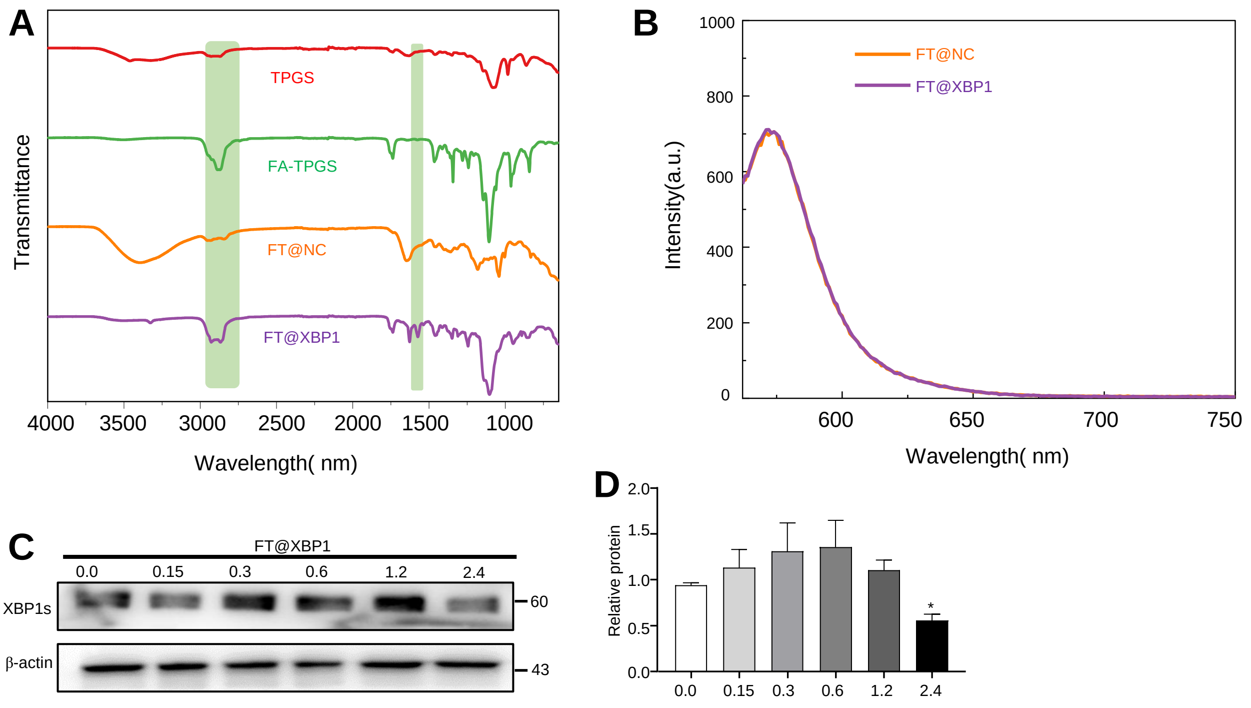

Figure S4. Characteristics and efficacy of the *XBP1* siRNA-loaded FA-TPGS. The Infrared spectrum (A) and (B) the fluorescence spectra of TPGS, FA-TPGS, FT@NC and FT@XBP1. (C) Western-blotting analyzed the expression of XBP1s in RAW 264.7 cells transfected with a series of FT@XBP1 and relative control, and (D) semi-quantitatively analyzed the band intensity. Data are presented as the means ± SD (error bar) of at least three independent experiments. **P*<0.05 as indicated.

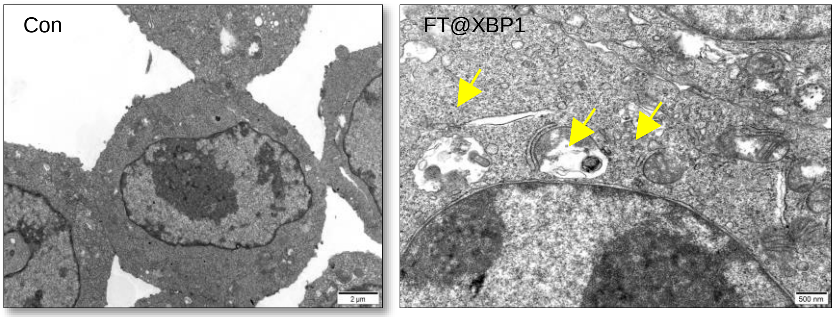

Figure S5. RAW 264.7 cells uptake FT@XBP1. Typical TEM observation of RAW 264.7 cells incubated with/without FT@XBP1 (scale bar=2μm/500nm).

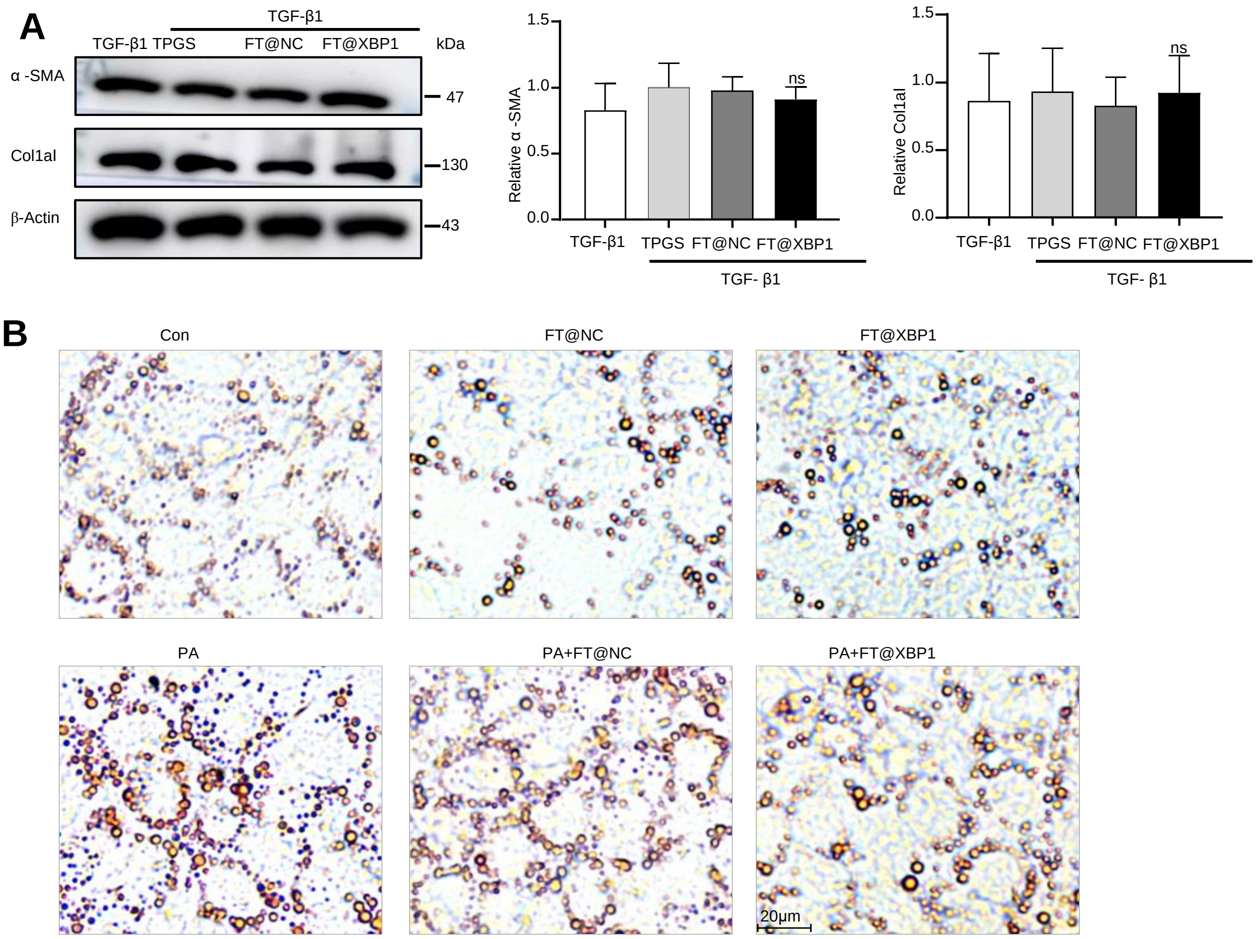

Figure S6. The impact of FT@XBP1 on JS-1 cells activation and AML-12 cells lipid deposition. (A) Western-blotting analyzed the expression of α-SMA and Col1αl in JS-1 cells treated with FT@NC and FT@XBP1, and the band intensity was semi-quantitatively analyzed. (B) Representative Oil red O staining images of AML-12 cells stimulated with palmitic acid or palmitic acid combination with FT@NC or FT@XBP1 (scale bar=50μm). Data are presented as the means ± SD (error bar) of at least three independent experiments.

.

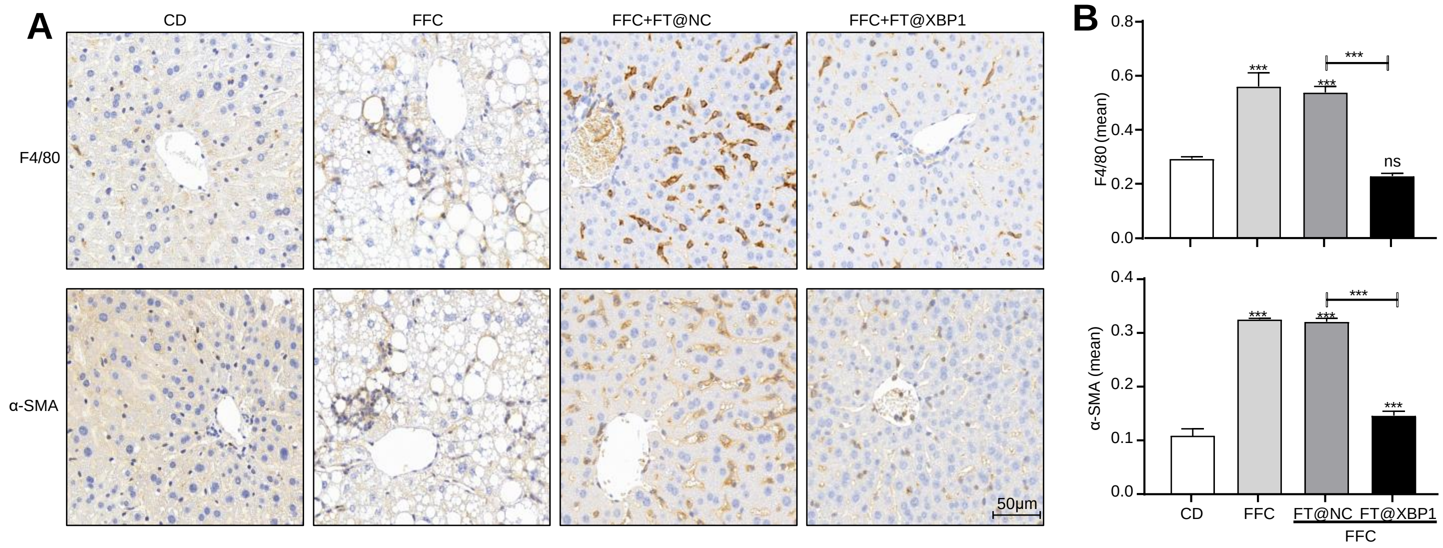

Figure S7. Macrophages co-localized with hepatic stellate cells. (A) Immunohistochemistry staining of F4/80 and α-SMA in NASH livers, and (B) quantitative analyzed (scale bar=50μm). Data are presented as the means ± SD (error bar) of at least three independent experiments. ***P<0.001 as indicated.

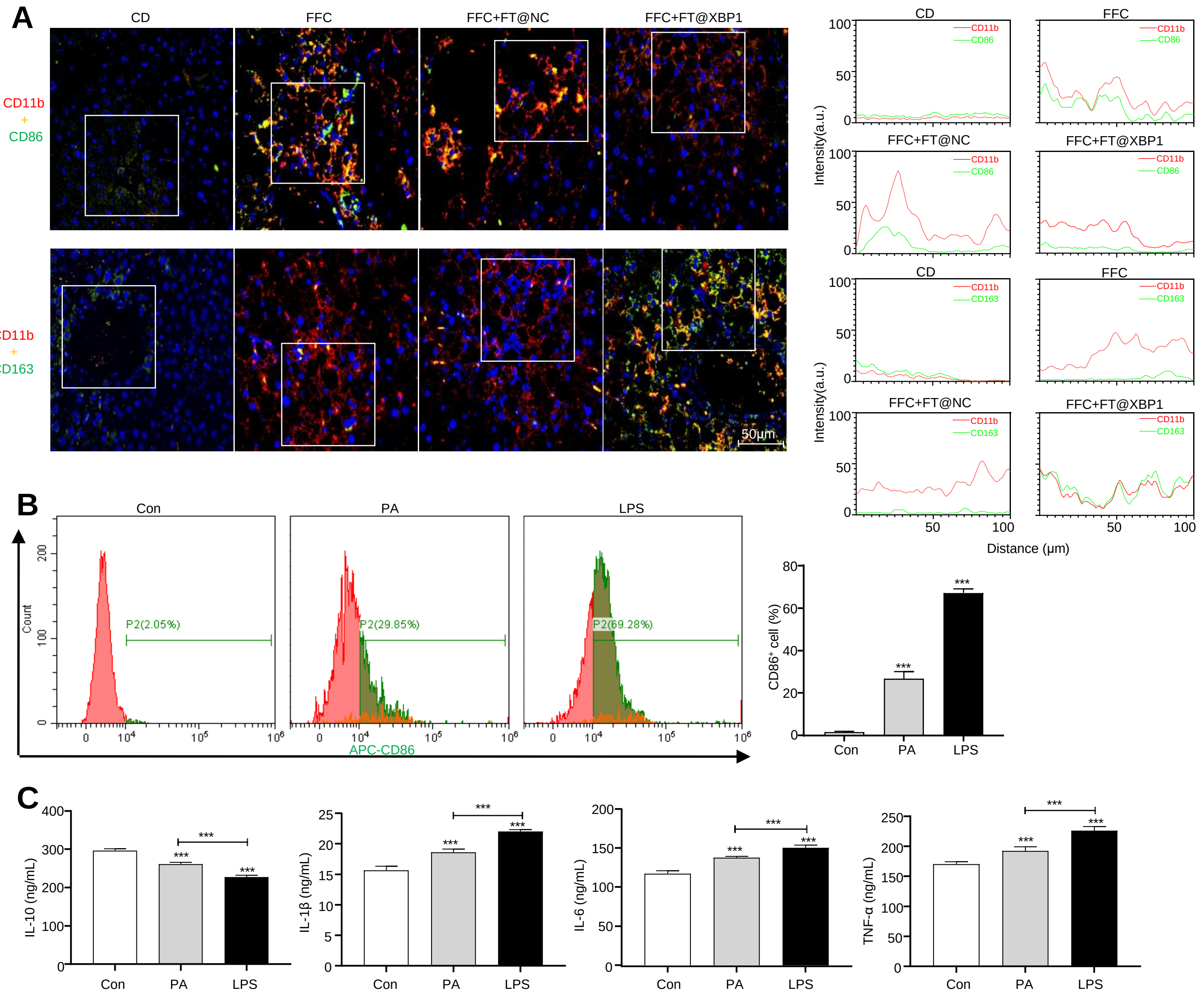

Figure S8. The impact of FT@XBP1 on macrophages polarization. (A) Representative immunofluorescence for dual staining CD11b and CD86 (the upper panel) or CD11b and CD163 (the lower panel) in FFC-diet induced MASH mice, and semi-quantitative analyzed (scale bar=50μm). (B) Flow cytometry detected the percentage of RAW 264.7 cells treated with palmitic acid and lipopolysaccharide. (C) The expression levels of IL-6, TNF-α, IL-1β and IL-10 in the cultural supernatants of RAW 264.7cells. Data are presented as the means ± SD (error bar) of at least three independent experiments. ***P<0.001 as indicated.

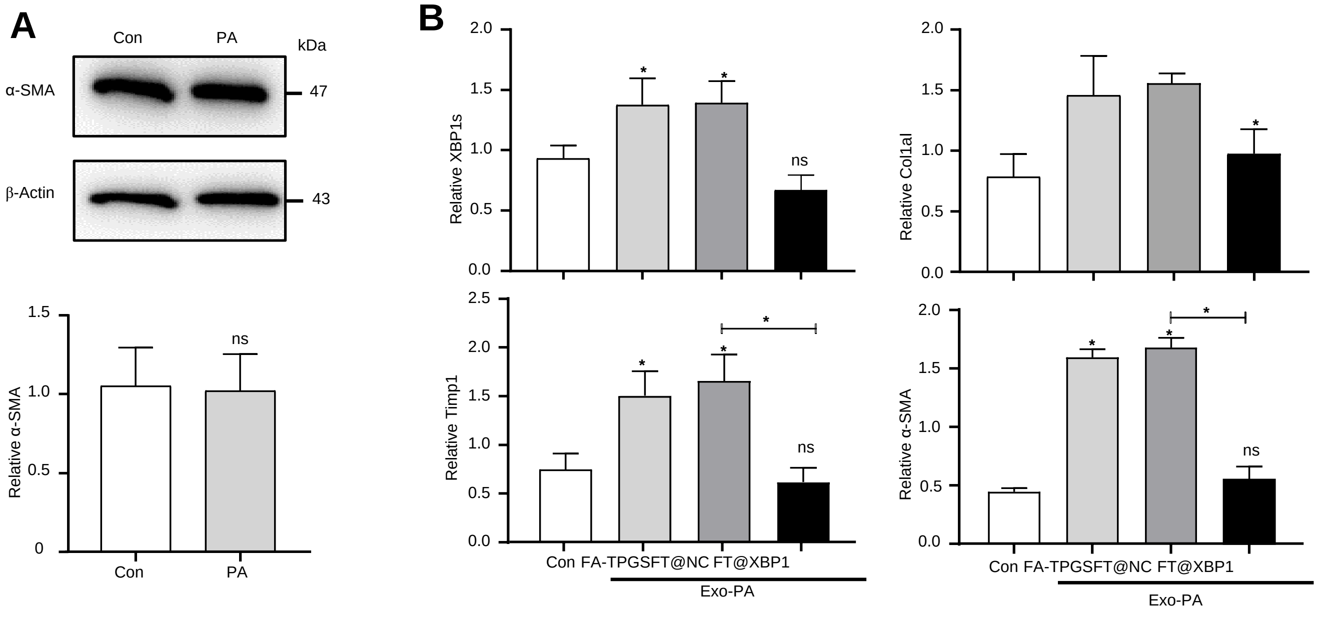

Figure S9. The impact of macrophages derived exosomes on JS-1 cells activation. (A) Western-blotting analyzed the expression of α-SMA in JS-1 cells treated with palmitic acid, and the band was quantitatively analyzed. (B) Semi-quantitatively analyzed the band intensity of XBP1s, α-SMA, Timp1 and Col1αI in Fig. 8F.
